# Low hypoxia tolerance as a determinant of the proinflammatory phenotype: a trained immunity perspective

**DOI:** 10.3389/fimmu.2026.1922856

**Published:** 2026-09-15

**Authors:** Dzhuliia Dzhalilova, Nikolai Fokichev, Olga Makarova

**Affiliations:** 1 Petrovsky National Research Centre of Surgery, Moscow, Russia; 2 Petrovsky Medical University, Moscow, Russia; 3 Faculty of Biology and Biotechnology, HSE University, Moscow, Russia

**Keywords:** high altitude, hypoxia tolerance, immune system, inflammation, mitochondria, oxidative stress, trained immunity

## Abstract

The connection between hypoxia and inflammation is well established at the local level, with HIF (hypoxia-inducible factor) playing a central role, which controls both the cellular response to oxygen deficiency and immune cell functioning. The literature contains extensive data regarding the roles of the transcription factors HIF, NF-κB (a key regulator of inflammatory processes) and NRF2 (a master regulator of antioxidant defense), as well as the roles of oxidative stress level and mitochondrial functional features, in the response to oxygen deficiency and the development of inflammation. Meanwhile, at the systemic level, organisms differ according to hypoxia tolerance, which may determine differences in the functioning of these factors. Hypoxia-susceptible organisms are characterized by higher oxidative stress levels, HIF and glycolysis activity, and distinct mitochondrial functional features. These characteristics may underlie a baseline proinflammatory phenotype and are associated with innate immune cell function and the activation of trained immunity. For instance, under the systemic hypoxia, particularly during the ascent to high altitude, some individuals exhibit increased blood proinflammatory cytokine levels, which is usually connected with high-altitude disease development. A critical unsolved question is whether low hypoxia tolerance is a cause or a consequence of the systemic proinflammatory phenotype, or whether both are driven by an upstream factor, such as mitochondrial dysfunction. This review summarizes current literature data on the connection between initial organism hypoxia tolerance and the proinflammatory phenotype and examines the role of oxidative stress, mitochondria, and trained immunity in these processes.

## 1 Introduction

Oxygen deficiency is a critical factor determining the development and severity of numerous pathological conditions, including cardiovascular, infectious, and inflammatory diseases and cancer (1, 2). At the same time, some evidence indicate that under certain circumstances, hypoxic exposure can exert beneficial effects (3, 4). For instance, lifespan is extended under low oxygen conditions in laboratory settings in model organisms such as *Caenorhabditis elegans* (5, 6). Furthermore, in some mammals, a connection was revealed between the habitat in hypoxic conditions and increased lifespan. For example, the naked mole rat, which inhabits burrows with reduced oxygen concentrations, and marine mammals, such as whales that hold their breath during deep dives, are characterized by the pronounced longevity (7, 8). Elderly people permanently residing at high altitudes generally live longer than those living at lower altitudes (9), although specific mechanisms remain to be established, since lifespan is influenced by many other factors besides hypoxia. Nevertheless, these observations highlight the potential role of hypoxia in modulating aging processes and suggest that adaptation to low-oxygen environments may contribute to longevity. However, hypoxic exposure effect depends on the dose and individual organism tolerance, a parameter that is often overlooked in studies due to the lack of standardized, non-invasive assessment methods (3, 4, 10).

Initial hypoxia tolerance is defined as an organism’s inherent capacity to maintain physiological function under conditions of reduced oxygen availability and to overcome hypoxic exposure through the metabolism reduction, oxygen consumption level decrease, and adaptation mechanisms to O_2_ deficiency activation, thereby avoiding pathological outcomes. Hypoxia tolerance is a complex, multisystem trait determined by genetic, epigenetic, and phenotypic factors, encompassing metabolic flexibility, cardiovascular and respiratory compensatory mechanisms, cellular stress responses, and immune regulation (11–16).

Hypoxic conditions can arise in diverse contexts—during diving, in burrows, at high altitude, in hibernation, and in clinical conditions such as stroke, emphysema, and asthma. Depending on the degree, duration, and other parameters (connection with pressure changes, tumor development, etc.), hypoxia is divided into many types that share common fundamental mechanisms but are also characterized by important pathophysiological differences. For example, in contrast to the continuous hypoxia, intermittent hypoxia, predominantly resulting from obesity-related transient upper airway obstruction during sleep, is considered an almost invariably pathological stimulus that induces maladaptive responses (17). Conversely, chronic exposure to intermittent hypoxic–hyperoxic periods at rest can be considered an efficient non-pharmacological strategy to improve physical and cognitive performance and reduce cardiometabolic risk factors (18). Responses to hypoxia can be acute (seconds to minutes) or chronic (hours to days). The distinction between organismal and environmental hypoxia is not always straightforward, and different hypoxia modeling approaches (normobaric vs. hypobaric, *in vivo* vs. *in vitro*, intermittent vs. continuous, etc.) provide effects that are difficult to compare directly. Nevertheless, different hypoxia types ultimately lead to cellular and tissue hypoxia, and the whole-organism hypoxia tolerance is determined by the tolerance of the most hypoxia-sensitive organs, particularly the brain (14, 15).

Hypoxic exposure is widely used to increase endurance in athletes, as well as for prevention and rehabilitation of various diseases (19). The protective effect is partly attributed to HIF-1α (hypoxia-inducible factor), which controls the response to oxygen deficiency and immune cell function; and NRF2 (nuclear factor erythroid 2-related factor 2), which governs antioxidant defense system activation (20). In addition to NRF2, nuclear factor-κB (NF-κB), which controls inflammation, is also a key transcription factor that can regulate cellular responses to oxidative stress. In addition to increasing antioxidant defense, hypoxic exposure affects innate immune cell functioning, which plays a key role in inflammation development, i.e., may exert an anti-inflammatory effect. At the cellular level, HIF, as well as NRF2, and NF-κB, are classified as key transcription factors (21). Hermes-Lima et al. (22) suggested that in hypoxia-tolerant animal species, interval hypoxic exposure induces a balanced increase in HIF, NRF2, and NF-κB levels, promoting antioxidant enzyme activation and posttranslational modifications that enhance antioxidant defense upon subsequent hypoxic exposures. Nevertheless, hypoxic exposure efficacy may depend on the basal activation levels of these transcription factors, which can vary significantly depending on the living altitude, age, and sex, as well as the individual initial hypoxia tolerance. In particular, hypoxia-susceptible organisms are characterized by a baseline proinflammatory phenotype; therefore, additional HIF, NRF2, and NF-κB stimulation in them may lead to adverse consequences. One potential manifestation of this proinflammatory phenotype in hypoxia-susceptible organisms may be the trained immunity development—the long-term functional reprogramming of innate immune cells induced by prior exposure to PAMPs (pathogen-associated molecular patterns) and/or DAMPs (damage-associated molecular patterns), which results in altered responses to subsequent stimuli through epigenetic and metabolic modifications (23–25).

It remains to be established whether low hypoxia tolerance is a cause of the systemic proinflammatory phenotype, whether a preexisting proinflammatory state predisposes to hypoxia susceptibility, or whether additional factors, such as structural and functional features of mitochondria, play a role in shaping both. In this review, we synthesized current evidence on the interconnection between the systemic hypoxia tolerance and inflammation, focusing on the transcription factors HIF, NF-κB, and NRF2, mitochondrial function, oxidative stress, and the trained immunity concept. We summarized literature data on the local interplay among these three key transcription factors activated in response to oxygen deficiency, oxidative stress, and inflammation, and on the variability of their activity, which depends on the initial organism hypoxia tolerance. Furthermore, we analyzed the connection between low initial organism hypoxia tolerance and proinflammatory phenotype, as well as the contribution of oxidative stress, mitochondrial dysfunction, and innate immune cells memory to these processes. This review focuses primarily on the systemic hypoxia, in particular hypobaric hypoxia encountered at high altitude and normobaric hypoxia used in experimental and therapeutic settings, with an emphasis on the individual organism hypoxia tolerance rather than on the hypoxic stimulus *per se*.

## 2 Molecular mechanisms of the cellular response to hypoxia: the interplay of HIF, NRF2, and NF-κB

### 2.1 HIF and oxidative stress activation in response to hypoxic exposure

The key transcription factor activated in response to hypoxic exposure is the HIF, which regulates more than 1,000 different genes. HIF regulates all organism-nucleated cells, including innate and adaptive immune cells; therefore, it plays an important role in the development of both physiological and pathological processes—inflammatory, reparative, tumor, etc. (26).

#### 2.1.1 HIF regulation mechanisms

Several main mechanisms of HIF activity regulation were identified—via prolyl hydroxylases (prolyl hydroxylase domain proteins—PHD1, PHD2, and PHD3) and factor inhibiting HIF—FIH-1. At sufficient oxygen concentration, the cytoplasmic HIF-α subunit is hydroxylated by PHDs at proline residues, which promotes proteasomal HIF-α degradation via pVHL (Von Hippel–Lindau) (27, 28). Hydroxylase activity depends on the tissue oxygen level: at low partial pressure or absence of O_2_, it is suppressed, which leads to the decreased HIF-α hydroxylation. As a result, the HIF-α subunit translocates to the nucleus, where it dimerizes with the HIF-β subunit. In the nucleus, the HIF-α/β dimer interacts with hypoxia response elements (HREs), which are located in oxygen-dependent gene promoters encoding proteins involved in systemic and cellular adaptation to hypoxia, such as erythropoietin and vascular endothelial growth factor (VEGF) (1, 29). Additionally, HIF transcriptional activity is regulated by FIH-1, which under sufficient oxygen conditions hydroxylates asparagine residues in the HIF-α subunit transactivation domain, thereby blocking binding to CBP/p300 transcription coactivators [CREB (cAMP-Response Element-Binding Protein)-Binding Protein/E1A Binding Protein p300] and preventing HIF-dependent gene transcription activation (30). Thus, depending on the oxygen level, proline and asparagine hydroxylation in HIF-α subunits negatively regulates their stability and HIF transcriptional activity regarding dependent genes.

#### 2.1.2 HIF isoforms temporal dynamics in hypoxic conditions

HIF family includes three α-subunit isoforms, HIF-1α, HIF-2α, and HIF-3α, the activation of which exhibits distinct temporal dynamics depending on the duration of hypoxic exposure. HIF-1α is expressed in all nucleated cells, whereas HIF-2α (EPAS, endothelial PAS) is predominantly in the vascular endothelium, kidney fibroblasts, hepatocytes, intestinal epithelial cells, cardiomyocytes, etc. (31–34). All HIF α-subunits dimerize with β- and bind to HREs. HIF-1α is mainly activated at the early stages (2–24 h) of the severe hypoxia or anoxia and promotes rapid glycolysis enzyme gene expression activation, whereas HIF-2α is activated during the prolonged hypoxia, and its activation persists for 48–72 h after the hypoxic exposure, promoting angiogenesis and erythropoiesis activation (35, 36). HIF-3α has many splice variants that may perform different functions, with some of them being negative regulators of HIF-1α and -2α subunits, as they dimerize with them and prevent their DNA-binding ability (37, 38). It is suggested that sequential activation of HIF-1α first, then HIF-2α, and ultimately HIF-3α allows cells to adapt to the prolonged hypoxia and promotes their survival (38).

#### 2.1.3 Mitochondrial ROS and HIF stabilization under hypoxia

Hypoxic exposure not only activates HIF but also promotes the production of reactive oxygen species (ROS), resulting in the oxidative stress. This phenomenon, termed “hypoxic oxidative stress,” is considered a key initiating event in the development of altitude-related pathologies (39). ROS can oxidize and damage mitochondrial membranes, as well as electron transport chain (ETC) enzymes, leading to oxidative phosphorylation (OXPHOS) suppression and ROS hyperproduction (40, 41). Restoring O_2_ flow to tissues can cause many concomitant disorders leading to the increased ROS production, mitochondrial Ca²^+^ overload, and, in extreme cases, their depolarization, cytochrome c release, and apoptosis (42–45).

Most experimental studies demonstrated that ROS levels increase rapidly under hypoxia due to several sources, such as mitochondria, membranes, cytoplasm, endoplasmic reticulum, lysosomes, and peroxisomes, the key of which are mitochondria (22, 46). It was demonstrated that Hep3B cells deficient in mitochondria failed to increase ROS during hypoxia (1% O_2_ during 1 h) (47). A major source of ROS is the mitochondrial ETC, the dysfunction of which leads to the formation of superoxide anion (O_2_^−^) (48). In human cells, superoxide levels transiently increase within 10 min of acute hypoxia (1% O_2_) (41).

In eukaryotes, electrons are transferred through the respiratory chain via mitochondrial enzyme complex I (nicotinamide adenine dinucleotide ubiquinone reductase), complex II (succinate ubiquinone oxidoreductase), and the integral membrane electron carrier CoQ (ubiquinone), mitochondrial enzyme complex III (cytochrome c reductase), electron carrier (cytochrome c), and finally through mitochondrial enzyme complex IV (cytochrome c oxidase) to molecular oxygen (46, 49, 50). Initially, mitochondrial complex III was identified as a source of short-term ROS production under hypoxia (1% O_2_ during 30–60 min) (51). The role of complexes I and II in ROS production was confirmed (42, 43, 52–54). In a recent study, healthy volunteers were subjected to a gradual, controlled reduction of inhaled oxygen to 10.5% (8-min exposure), the effect of acute hypoxia on mitochondrial respiration and ROS production in peripheral blood mononuclear cells was determined. Such hypoxia exposure to the organism disrupts mitochondrial respiratory chain functioning, rapidly reducing complex II activity in combination with increased succinate receptor 1 (SUCNR1) activity, and expression activation of HIF-1α and some genes involved in inflammatory processes, such as signal transducer and activator of transcription 3 (STAT3), chemokine ligand-9 (CXCL9), and interferon-stimulated gene 15 (ISG15). These results indicate the role of mitochondria in inflammation development under hypoxic exposure to the organism and the need for further studies on complex II activity influence on inflammatory and metabolic processes observed during hypoxia (55).

Data regarding the role of ROS in HIF stabilization are contradictory. On the one hand, some studies suggest that HIF-1α protein stabilization under hypoxia occurs independently from the mitochondrial ROS production, and that the cellular response to oxygen deficiency may be controlled by changes in mitochondrial respiration through regulation of oxygen availability to cells (56, 57). On the other hand, numerous studies demonstrated that mitochondrial ROS generated during hypoxia function as signaling molecules, and PHDs as ROS sensors, i.e., mitochondrial ROS production is necessary for HIF-1α stabilization under hypoxia (58–60). Other studies revealed that mitochondrial ROS, whose levels increase during hypoxia, promote HIF activation (47, 61). ROS can lead to HIF activation through PHD-independent pathways, for example, through FIH inhibition and NF-κB activation, which can also stimulate HIF (59, 62–64). Furthermore, oxidant-induced GSH Cys520 adduct accumulation in the HIF-1α protein promotes its stabilization (65). All these data collectively indicate that HIF activation occurs in response to oxidative stress (66), and hypoxia and oxidative stress are interconnected rather than independent factors.

Under the prolonged hypoxia, excessive ROS accumulation may be dangerous for cells; therefore, mechanisms for reducing ROS production and neutralizing them are being activated in cells. For instance, HIF-1α activation promotes mitochondrial protection from oxidative stress by increasing the PDK1 (pyruvate dehydrogenase kinase 1) levels, thereby activating mitochondria-independent metabolism. Furthermore, HIF-1α increases electron transfer efficiency and promotes ROS accumulation reduction through the complex I inhibition (67, 68). In various animal hypoxic exposure models, increased antioxidant activity such as glutathione peroxidase (GPX) and superoxide dismutase (SOD) was detected (22).

Thus, under hypoxia, cells need both to maintain HIF isoform stabilization for successful adaptation and to prevent ROS-induced organelle damage. The balance of these processes largely depends on the NRF2, data on which activation under hypoxia are quite contradictory and likely depend on the exposure duration and severity.

### 2.2 NRF2 and antioxidant activation in response to the hypoxic exposure, as well as link with the HIF

The main transcription factor regulating antioxidant defense and detoxification, and providing cell adaptation and survival under oxidative stress caused by both hyperoxia and hypoxia, is NRF2 (69). Hyperoxia—a condition opposite to hypoxia—causes membrane oxidation, disruption of cellular organelle function, and, ultimately, cell death. In response, the expression of genes involved in ROS inactivation and damaged cells recovery increases.

#### 2.2.1 NRF2-Keap1 regulation

In the absence of oxidative stress, NRF2 bounds to the cytosolic protein Keap1 (Kelch-like ECH-associated protein 1) and constantly ubiquitinates and degrades. In case oxidative stress occurs, key Keap1 cysteines are modified, leading to conformational changes that prevent the NRF2 degradation. Free NRF2 accumulates and translocates to the nucleus, where it heterodimerizes with sMAF proteins. This complex binds to antioxidant response elements (AREs; 5′-A/GTGACnnnGC-3′) and initiates the transcription of more than 200 genes, encoding antioxidant enzymes, such as quinone oxidoreductase 1 (NQO1), aldo-keto reductases, glutathione (GSH) S-transferases, and UDP glucuronosyltransferases, proteasomal proteins, and xenobiotic efflux systems. Furthermore, NRF2 influences on the heme and iron metabolism-related genes, such as HO-1 and ferritin (70). This regulatory mechanism is crucial for maintaining the redox balance in cells and protecting against oxidative stress-related damage. Thus, NRF2 activation protects cells from ROS damage (71, 72). It is suggested to use the NRF2 activation, for example with dimethyl fumarate, as a therapeutic strategy to reduce oxidative stress levels in inflammatory and neurodegenerative diseases such as multiple sclerosis and Parkinson’s disease (73, 74).

#### 2.2.2 NRF2 in hypoxia

Despite NRF2’s role in hyperoxia, data regarding its activity under hypoxia-induced oxidative stress are contradictory. Probably, NRF2 activation largely depends on the hypoxic exposure duration and severity, as well as individual organism hypoxia tolerance. NRF2 was demonstrated to play a crucial role in stimulating the organism’s defense systems and potentially respond to oxygen deficiency during the hypoxic training among athletes. Acute hypoxia exposure may cause the NRF2-ARE pathway activation, with the response severity correlating with hypoxic exposure duration, and HIF-1α activation is involved in enhancing NRF2-mediated antioxidant responses in skeletal muscles (20). Other studies on tumor cells also demonstrated a positive connection between HIF and NRF2 (75). Knockdown of the gene encoding NRF2 is sufficient to reduce HIF-1α activity at the posttranslational level, suggesting that NRF2 or its downstream targets play a role in regulating PHDs activity (76, 77). The NRF2 inhibitor brusatol was demonstrated to reduce the HIF-1α protein level in colon cancer cells by promoting its proteasomal degradation, thereby suppressing glucose uptake. These findings highlighted the therapeutic potential of the NRF2 inhibitors (76, 77).

Despite temporary ROS and antioxidant activity increase under hypoxia, in some studies the sustained NRF2 activation was not detected. Under certain experimental conditions or depending on cell type, the HIF and NRF2 connection revealed the reverse dependence. It was demonstrated that HIF-1α activation reduces NRF2 transcriptional activity in ischemic mouse kidneys and vascular endothelial cells (78, 79). Treatment of human endothelial cells with andrographolide—the main bioactive component of the medicinal plant *Andrographis paniculata*, which has pronounced anti-inflammatory effects—suppresses HIF-1α, and this process involves NRF2 activation (80). NRF2 activator application was considered as a therapeutic method for acute mountain sickness (AMS) therapy (81). The HIF–NRF2 interaction was discussed in more details in a recent review (75).

Furthermore, literature data indicate that during *in vivo* hypoxic exposure at 5,000 m, both rat retinal NRF2 mRNA and protein levels remain decreased during hypobaric hypoxia (82). During *in vitro* acute (2 h) and chronic (12 h) 1% O_2_ exposure in human normal renal proximal tubular epithelial cells HK-2 and primary murine kidney epithelial (pMKE) cells, the NRF2 protein level decreased, whereas the HIF-1α level increased (83). Therefore, it was suggested that other hypoxia-activated transcription factors, such as NF-κB, may contribute to the upregulation of antioxidant proteins (22).

Thus, under hypoxia, at the cellular level depending on the exposure extent and duration, HIF-contributed adaptation was predominantly activated, as well as the NRF2-dependent antioxidant defense and NF-κB-dependent inflammatory responses, and the resulting outcome of this interaction (successful adaptation or its disruption) critically depended on the balance between them.

### 2.3 NF-κB activation in response to hypoxic exposure, as well as link with HIF and NRF2

On the one hand, under hypoxia, HIF, NF-κB, and NRF2 activation is considered the main regulatory mechanism for maintaining tissue and organ homeostasis, attenuating oxidative damage, inflammation, and other physiological cellular and systemic reactions, which result in successful cell adaptive response (**Figure 1**). Examples of successful adaptation include substantial hypoxia-induced angiogenesis in the coronary circulation, which enhances myocardial O_2_ transport under hypoxic conditions. In addition, in the lung of natives and long-term high-altitude residents and in young animals exposed to the long-term hypoxia, alveolar-capillary proliferation occurs, inducing substantial increases in the pulmonary diffusion surface as manifested in marked increases in diffusing capacity both at rest and during the exercise (84). On the other hand, in the case of disruption of the balance of these factors’ activation, pathophysiological reactions development – maladaptive responses – may occur (12, 17, 85). Examples of maladaptive responses include the AMS development under acute hypoxia and chronic mountain sickness (CMS) under chronic hypoxia. CMS shares many mechanisms with diseases commonly detected at sea level in which hypoxia plays an important role, such as chronic obstructive pulmonary disease, sleep apnea, and tumors (86). Furthermore, maladaptive response can be detected in case of intermittent hypoxia exposure. The maladaptive side of intermittent hypoxia may be observed in the cardiovascular, metabolic, and cognitive consequences of obstructive sleep apnea, which include excessive oxidative stress and inflammation, increased cerebral and systemic vascular resistance, systemic and pulmonary hypertension, and elevated insulin resistance (84, 87). The balance between adaptive and maladaptive responses apparently primarily depends on the organism’s ability to respond and activate adaptive mechanisms to hypoxia. These terms are also used in the immune system context. Thus, Halper-Stromberg A. and Jabri B. (88). suggest that under the influence of various factors, immune cells adapt and acquire an individual immune identity, reflecting the overlay between the genetic predisposition and the antigenic and environmental exposures of each individual. While some aspects of this immune shaping are natural consequences of immune maturation over the time, others are maladaptive and predispose to irreversible pathology. In **Figure 1**, under the terms adaptive and maladaptive responses, we primarily mean the immune system response, as well as the absence/presence of inflammatory reactions in response to hypoxic exposure.

**Figure 1:**
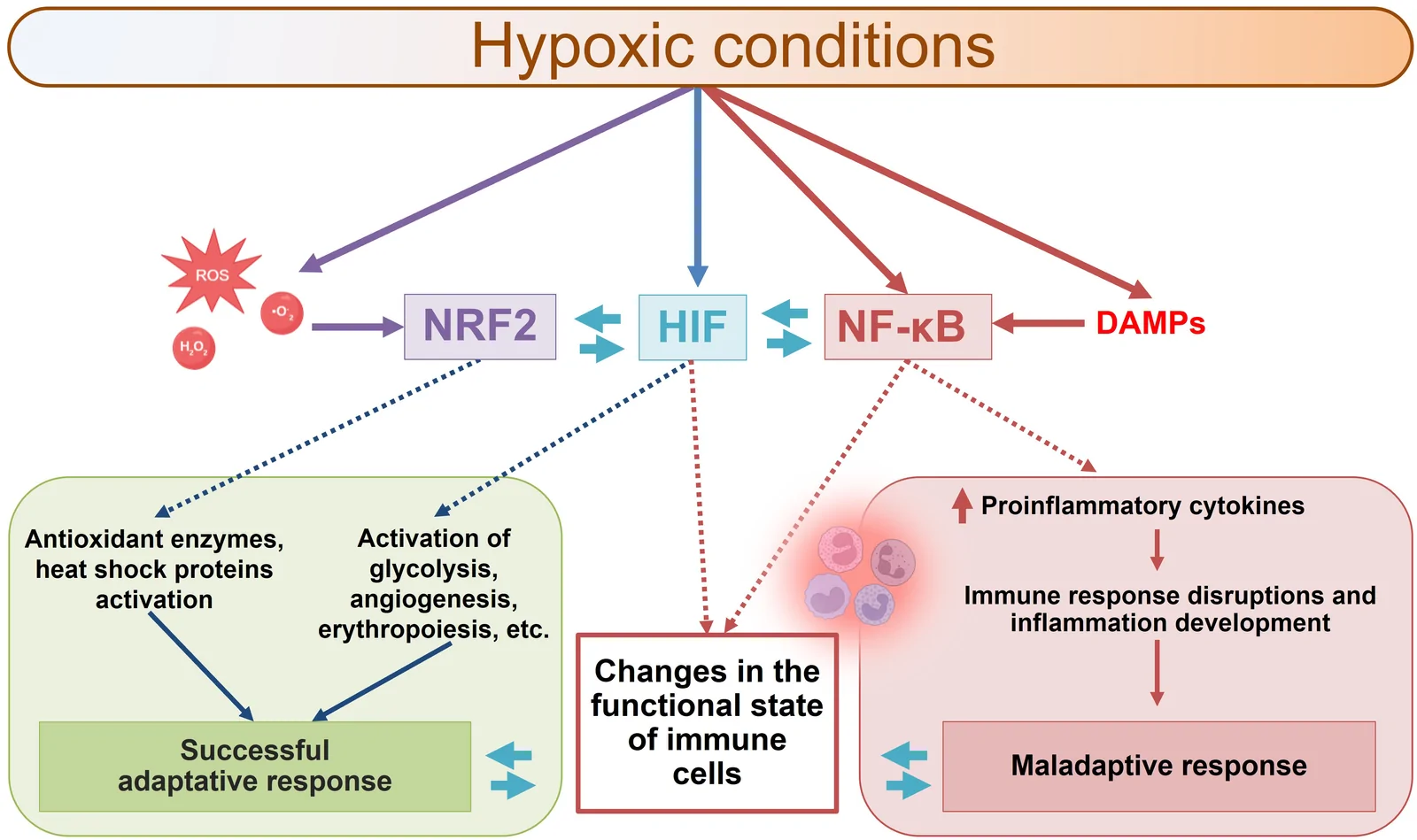
Transcription factors HIF, NRF2, and NF-κB interconnection and their role in regulating the response to hypoxia. HIF isoforms are activated during hypoxia and regulate genes providing adaptation to it (activation of glycolysis, angiogenesis, erythropoiesis, etc.). Moreover, HIF isoforms cause the changes in immune cells’ functional state. NRF2 is activated in response to oxidative stress and regulates antioxidant defense. NF-κB is activated in response to hypoxia, oxidative stress, and DAMP release, regulates antioxidant defense, and also promotes proinflammatory cytokine expression increase and causes changes in immune cells’ functional state. Balance between these pathways determines the outcome (successful adaptative response or maladaptive response).

#### 2.3.1 Hypoxia-induced NF-κB activation

The transcription factors NF-κB are hetero- or homodimers of proteins—NF-κB1 (or p50), NF-κB2 (or p52), RelA (or p65), RelB, and c-Rel—and can be activated by canonical and non-canonical pathways. NF-κB polymorphisms underlie development of many diseases, including autoimmune diseases, neurodegenerative diseases, and cancer (89). Emphasizing the central role of oxidative stress in inflammation, Valacchi et al. (2018) suggested the term “OxInflammation” (90). However, a validated oxidative stress assessment method was not yet developed (91, 92).

Hypoxic conditions promote inflammation through oxidative stress, DAMPs production, and PHD inactivation, which are connected with NF-κB inhibitors. Hypoxia activates NF-κB-dependent gene transcription and increases this pathway sensitivity to proinflammatory stimuli, such as cytokines, by reducing the hydroxylation of key NF-κB regulators—IKK (93). Both IKKα and IKKβ contain an evolutionarily conserved consensus motif in their kinase domains that is hydroxylated by PHD enzymes. Thus, the same hydroxylases that regulate the oxygen sensitive pathway and promote ubiquitin-dependent HIF-1α degradation, control the kinase complex activity responsible for NF-κB regulation and play a determining role in its response to hypoxia (93, 94). Under normoxia, IKKβ appears to be hydroxylated by PHD1 and PHD2, leading to the suppression of its enzymatic activity and NF-κB inhibition. Under hypoxia, IKKβ hydroxylation by PHDs is suppressed, causing NF-κB activation and proinflammatory and antiapoptotic gene expression (62, 93). Thus, under oxygen deficiency, hydroxylase inhibition of NF-κB pathway components occurs, which may lead to its activation and represent a critical point of the connection between hypoxia and inflammation (93).

#### 2.3.2 HIF proinflammatory and anti-inflammatory roles

The direct role of HIF in NF-κB activation remains insufficiently understood. Under inflammatory conditions, HIF-1α can limit NF-κB transcriptional activity *in vitro* and *in vivo* (95). Studies in mammalian tumor cell lines demonstrated that, in the absence of HIF-1α, NF-κB activity is increased. An evolutionarily conserved negative feedback mechanism was discovered through which HIF can regulate NF-κB by IKK- and CDK6 (cell division protein kinase 6)-dependent pathways (95). In the experimental osteoarthritis model, HIF-1α suppressed the NF-κB activation, indicating its anti-inflammatory and chondroprotective effects (96). These findings support the fact that HIF activation may also exert an anti-inflammatory effect. In this case, HIF is one of the negative feedback components important for limiting NF-κB activity (95). Apparently, HIF activation can be both proinflammatory and anti-inflammatory depending on conditions, cell type—tumor or non-tumor, epithelial or myeloid—and organism hypoxia tolerance (95–98).

For instance, in local inflammation, HIF-1 activation leads mainly to anti-inflammatory effects; in contrast, in systemic inflammatory response, it leads to proinflammatory outcomes. Epithelial cells play a key role as the first line of defense against microorganisms in tissues such as the lungs, skin, and gastrointestinal tract. Experiments demonstrated the protective role of HIF-1 in infectious inflammation foci for different cell types, for example, intestinal epithelial cells and keratinocytes (99–101). HIF-1α upregulates many barrier-protective genes, including those encoding mucins and β-defensins, and reduces epithelial cell apoptosis (102). Enhanced inflammation was also demonstrated in a DSS-induced colitis model in mice with myeloid HIF-1α and HIF-2α knockout, and the protective role of predominantly HIF-1α in myeloid cells during inflammatory bowel diseases is discussed (103). In contrast, reduced inflammatory response was established in animals with myeloid HIF-1α knockout in an acute skin inflammation model (104). Another study established opposing roles for two isoforms: in myeloid cell *Hif1a* knockout, colitis course improved, whereas *Hif2a* knockout, on the contrary, worsened, due to increased neutrophil recruitment to the deeper colon layers, leading to higher *Il6, Ifng, Cd11c, Cd4*, and *Cd8* expression in the colon (105). HIF-1α knockout in dendritic cells showed increased clinical symptoms of DSS-induced colitis in mice with a lower number of Tregs (106). Despite the HIF-1α-protective function in intestinal epithelial cells during local inflammation, it may play a negative role in inflammation with pronounced systemic manifestations, particularly in case of sepsis. In a gram-positive infection model induced by lipoteichoic acid and peptidoglycan, HIF-1α deficient in myeloid cells mice exhibited reduced tissue damage and high survival rates (107). HIF-1α knockout in myeloid cells led to increased mouse survival in lipopolysaccharide (LPS)-induced sepsis, decreased serum proinflammatory cytokine levels, and increased sepsis-protective IL-10, which exerts immunosuppressive and anti-inflammatory effects (108). At the same time, in rats during sepsis, intestinal HIF-1α activation promoted epithelial barrier protection by inhibiting inflammation and oxidative damage (109). Recent evidence indicates that HIF‐2α plays an inhibitory role in many proinflammatory responses, which is discussed in detail in the review (110). Thus, HIF activation effect in various inflammatory diseases significantly depends on cell type and process prevalence. Further studies are needed, especially on HIF-3’s role in inflammatory process development.

HIF isoforms are widely expressed and found in almost all innate and adaptive immune cell populations, including macrophages, neutrophils, dendritic cells, and lymphocytes, which largely determines the severity of the inflammatory disease course. Both NF-κB and HIF-1α can be considered as major regulators of inflammatory gene expression involved in many diseases’ development (111). Furthermore, it was established that HIF-1β is necessary for full NF-κB activation in cells after various stimuli (112).

#### 2.3.3 NF-κB-dependent HIF activation

NF-κB and HIF are considered major participants in the inflammatory and innate immune response, which can be proinflammatory or anti-inflammatory depending on the cell type (62, 95, 113–115). NF-κB influences HIF-1α activation both under normoxia and hypoxia, and in response to inflammatory stimuli, and also controls *HIF1A* gene expression levels and HIF-1β protein content (62–64). In the proximal part of the *HIF1A* gene promoter, at −197/−188 base pairs, a conserved active NF-κB-binding site is contained, indicating that the connection between these two pathways is widespread among various organism species (62–64, 116, 117). In addition to induction through the NF-κB-dependent pathway, during inflammation, functional HIF-1α activation is carried out by proinflammatory cytokines—IL-1β and TNF-α, as well as other factors—ROS, NO, and PI3K (118). Increased ROS production during the inflammation leads to PHD inhibition and HIF-1α activation (119, 120). It should be noted that the conditions under which HIF-1α is induced (hypoxia or inflammation) affect the outcome of its activation. When HIF-1α is activated in response to hypoxia, it enhances the transcription of target genes that promote adaptation to oxygen deficiency. When induced through the NF-κB-dependent pathway, HIF-1α predominantly drives the proinflammatory cytokine gene expression (121).

#### 2.3.4 NRF2-NF-κB interplay

In response to the oxidative stress and inflammation, NRF2 is considered as another important transcription factor involved in maintaining redox homeostasis and regulating coordinated induction of antioxidant and anti-inflammatory genes (70, 122, 123). Obviously, in response to hypoxic exposure, there is extensive crosstalk between various transcription factors, including the connection between HIF and NF-κB (62, 111), as well as NRF2 and NF-κB (124). The molecular mechanisms underlying the NRF2 and NF-κB interaction appear to be cell- and tissue-type-dependent and remain incompletely characterized (125). On the one hand, NRF2 can regulate inflammatory processes and negatively influence NF-κB through direct and indirect mechanisms. NRF2 directly inhibits oxidative stress-mediated NF-κB activation by reducing intracellular ROS levels and enhancing antioxidant gene expression, which blocks proinflammatory cytokine transcription, including IL-1β and IL-6. Furthermore, NRF2 prevents IκB-α inhibitor proteasomal degradation, which blocks NF-κB nuclear translocation (125–127). On the other hand, NF-κB reduces free CBP levels, which is an NRF2 transcriptional coactivator, by competing with the CBP CH1-KIX domain, and in addition prevents NRF2 binding to ARE by recruiting the corepressor histone deacetylase 3 (HDAC3) (128).

Although the local interplay among HIF, NF-κB, and NRF2 is quite well characterized, the dynamics and functional consequences of their induction in different cell types both under normoxia and under systemic hypoxia exposure—in highlanders, as well as in organisms with different oxygen deficiency tolerance—remain unclear. The fundamental unsolved problem is whether the characterized molecular mechanisms are universal, or whether differences exist in the basal activity of these pathways that are associated with initial individual organism hypoxia tolerance and the proinflammatory phenotype.

## 3 Interindividual differences in hypoxia tolerance and its association with HIF, NRF2, and NF-κB activation

High-altitude conditions provide an adequate and suitable model for systemic-level investigation of the interaction of all three factors—HIF, NRF2, and NF-κB—since oxygen deficiency and activation of antioxidant system, as well as inflammatory processes are observed at altitude. However, there are interspecies and intraspecies differences in response to hypoxic exposure, connected including the activation features of these three transcription factors and individual tolerance. Organism hypoxia tolerance variability arose in parallel with the aerobic metabolism evolution, as different organisms developed adaptation mechanisms to reduce oxygen availability. Different species are adapted differently to decreased O_2_ availability; in humans, there are individual features of physiological responses to hypoxia (129). For instance, in athletes, the individual responses to high-altitude training vary significantly (130). While some athletes note significant performance improvement, others demonstrate minimal improvements or even its decline (131, 132). Training regimens should consider individual organism hypoxia tolerance; however, non-invasive and highly reproducible methods for its assessment without hypoxic exposure were still not developed.

### 3.1 Hypoxia tolerance determination approaches

Research aimed at understanding the individual tolerance to oxygen deficiency and at developing methods for its determination is actively ongoing. One of the most common methods is the model reproducing hypobaric hypoxia conditions in decompression chambers, into which volunteers, pilots, astronauts, and experimental animals are placed (10–13, 133–144) for examination. However, the predominant method for assessing human hypoxia tolerance is staying under conditions corresponding to an altitude of several thousand meters and dividing into tolerant and susceptible depending on their susceptibility to AMS, high-altitude pulmonary (HAPE), and cerebral edema (HACE) (12, 139, 143, 144). In experimental studies, animals were stratified into hypoxia-tolerant and hypoxia-susceptible groups based on their “gasping time” (time until respiratory disturbances, asphyxia signs, and lateral position assumption) in a decompression chamber simulating hypobaric hypoxia. The “gasping time” of tolerant animals typically differed from the susceptible ones by at least threefold. Based on this assessment, a substantial proportion of animals was characterized as moderately hypoxia-tolerant (40–58%), whereas the proportions of tolerant and susceptible animals range from 20%–42% and 18%–40%, respectively. Moderately hypoxia-tolerant animals are generally not used in subsequent experiments (13, 133, 142).

### 3.2 Genetic and epigenetic determinants of hypoxia tolerance

Hypoxia tolerance is determined by genetic, epigenetic, and phenotypic organism properties: the origin of its energy metabolism, the efficiency of its regulatory mechanisms, and the capacity of these mechanisms to reorganize and adapt to hypoxic conditions while maintaining vital functions (11–13, 133). It was demonstrated that the hypoxia tolerance phenotype in *Drosophila melanogaster* selected under oxygen deficiency conditions is heritable (145). These flies also exhibited several phenotypic changes, including improved mobility under severe hypoxia, significant reduction in body mass and size, decrease in number and size of wing cells, reduced mitochondrial oxygen consumption rate and ROS formation, and mitochondrial ultrastructure changes.

In Ragsdale et al.’s (146) study, epigenetic mechanisms providing hypoxia tolerance were established. Short-term 14-day hypoxic exposure of male *Danio rerio* led to increased hypoxia tolerance in their offspring that had not been subjected to oxygen deficiency. Offspring exhibited a marked (more than 6-fold) increase in hemoglobin gene *hbaa1* and *hbz* expression, which correlated with their phenotypic hypoxia tolerance (146). At the same time, the authors did not detect differential methylation in these genes’ DNA loci; therefore, they suggest that other epigenetic mechanisms, such as small non-coding RNA changes or histone modifications, are responsible for transmitting the “epigenetic memory” of effective hypoxic adaptation (146). The role of epigenetic factors in adaptation to hypoxia was discussed in detail in the review (85).

### 3.3 HIF and dependent gene activity in organisms with different hypoxia tolerance

HIF and dependent gene activity differs in organisms with different hypoxia tolerance, as well as in those living on plains and in high-altitude conditions. In work (147), the presence of variations in the HIF system response to hypoxic exposure at the cellular level between healthy individuals was investigated. In the absence of hypoxic stimulus, significant differences in basal expression of HIF-regulated genes in peripheral blood lymphocytes were observed. Under eight different partial oxygen pressure levels (ranging from 20% to 0.1%), significant interindividual differences in the degree of these genes’ expression induction were also observed. The expression profiles of the four studied genes—*VEGF*, aldolase C (*ALDC*), adrenomedullin (*AM*), and prolyl-4-hydroxylase α1 (*P4HA1*)—in lymphocytes were related to oxygen tension in a non-linear manner. Unlike the basal expression levels, a common pattern of hypoxia-induced gene expression was observed among genes: if one gene was strongly induced by hypoxia in a particular individual, this generally also occurred with all the other dependent genes in that individual. It was suggested that organisms differ significantly in a factor common to all studied genes regarding the induction of their expression by hypoxia. The common nature of variability of HIF-regulated genes *VEGF*, *ALDC*, *AM*, and *P4HA1* suggests that the source of this variation lies within the HIF family itself (147).

Organisms inhabiting high-altitude environments exhibit structural changes in the genes encoding HIF and its downstream targets (148), allowing them to adapt to low oxygen availability. Advances in genome sequencing confirmed the role of genetic predisposition to high-altitude diseases. However, current data on how exactly genetic variations affect hypoxia susceptibility remain limited.

In studies on high-altitude populations, polymorphisms in both nuclear and mitochondrial genes particularly in residents of the Andes, Ethiopia, and Tibet were identified (149), providing adaptation to chronic hypoxia, including changes in erythrocytes, hemoglobin content, angiogenesis, vasoregulation, and immune response (150, 151). Polymorphisms of the *EPAS1* (endothelial PAS domain protein 1) gene encoding HIF-2α and *EGLN1* (Egl-9 family hypoxia-inducible factor 1) encoding PHD2 were identified, including rs2278753, rs1562451, rs7557402, rs149594770, rs9679290, rs11125070, rs2034327, rs13003074, rs6741821, rs11122281, rs2808611, rs540817, rs2790873, and rs2275279, associated with populations living at high altitudes. The presence of such SNPs in hypoxia-response genes leads to attenuated HIF activity and a dampened erythropoietic response, thereby reducing the risk of CMS development during permanent residence in the mountains (152). These features provide insight into the organism’s adaptive responses to chronic hypoxia and serve as a model for new treatment methods for various diseases based on correction of individual oxygen deficiency tolerance research and development (11).

Polymorphisms of *EPAS1*, *EGLN1*, *VEGFA*, and other genes associated with high and low AMS risk were identified (153). For example, the presence of four SNPs (rs13419896, rs4953348, rs4953354, and rs6756667) was associated with the increased AMS risk in sea level residents, especially *EPAS1* rs6756667 (154, 155). Collectively, these results indicated that *EPAS1* polymorphisms play an important role in AMS development, and HIF-2, responsible for implementing the cellular response to chronic hypoxia, may be a therapeutic target in oxygen deficiency-associated diseases.

Epigenetic mechanisms of high-altitude adaptation were established. In Tibetans, key HIF pathway genes such as *EPAS1* and *EGLN1* have unique methylation patterns driven by natural selection, increasing hypoxia tolerance in comparison with other populations (e.g., Han) (156, 157). A similar HIF pathway methylation regulation pattern was also observed in Andean high-altitude adaptation studies (158, 159). Nonetheless, abnormal methylation patterns may disrupt HIF pathway homeostasis, leading to acute and chronic high-altitude diseases (85).

According to experimental studies, hypoxia-tolerant and hypoxia-susceptible animals differ in HIF-1α content in the liver and colon (**Table 1**) under normoxia and in response to hypoxic and inflammatory stimuli (133, 142, 173). In susceptible rats under normoxic conditions, its content in the neocortex is 1.7 times higher than in tolerant rats (133). It should be noted that it is HIF-1α (not HIF-2α) that stimulates glycolytic gene expression (33); therefore, high baseline HIF-1α expression in organisms susceptible to hypoxia may likely determine initially high glycolysis levels. Constitutively elevated HIF-1α and glycolysis levels may be associated with high oxidative stress levels, as well as with mitochondrial functional features—the main cellular ROS source—and NRF2 activity.

**Table 1:** Changes in factors regulating response to hypoxia, inflammation, and oxidative stress activity at high-altitude ascent in healthy volunteers, and differences of hypoxia-tolerant and hypoxia-susceptible animals according to experimental data.

Model/conditions	Group characteristics	HIF activity	Oxidative stress and mitochondria	Inflammatory markers	Reference
Human studies					
High-altitude (study A—3,458 m, 68 h; study B—3,647 m, 3 overnight stays and 22 h stay at 4,559 m)	Study A—n=12 (1 females, 11 males, age 28 ± 2 years) Study B—n=10 (3 females, 7 males, age 32 ± 5 years)	–	–	In both studies, participants demonstrated AMS symptoms, with increased IL-6, IL-1ra, and CRP in plasma	(160)
High-altitude (4,350 m, 4 days)	n=10, males, age 32 ± 5 years	–	–	Subjects had mostly light AMS symptoms with the increased serum IL-6 level, whereas IL-1β, IL-1ra, TNF-α, and CRP levels remained unchanged	(161)
High-altitude (3,800 m, 3 days)	n=15 (5 females, age 26 ± 5 years, 10 males, age 25 ± 4 years)	Increased *HIF1A* expression in blood	Increased *NFE2L2* expression in blood	Increased *HMGB1*, *S100A8*, *TLR4*, *LY96*, and *IL8* expression in blood	(162)
High-altitude ascent (from 1,289 to 7,546 m, 16 days)	n=11, 9 males (18–53 years) and 2 females (33 and 42 years)	Increased *HIF1A* expression in monocytes, B-lymphocytes, T-lymphocytes, and NK cells	Increased *SOD2, PRDX4, TXN*, and *NFE2L2* expression in monocytes and plasma cells, *LDHA* and *GAPDH* were significantly elevated in CD8+ effector memory T cells and plasma B cells	Increased *IL1B, TNF*, and *NFKB1* expression in monocytes and dendritic cells; neutrophils and monocytes number, plasma CRP level	(163)
High-altitude (3,830 m, 72 h)	n=15, 11 males, 4 females, age 39 ± 10.2 years	Increased *HIF1A* and *EPAS1* expression in white blood cells	Increased *NFE2L2* expression in white blood cells, ROS production, TBARS (thiobarbituric acid reactive substances), 8-isoprostanes (8-isoPGF2α), 8-hydroxy-2′ -deoxyguanosine (8-OHdG), protein oxidative carbonylation concentrations, and decreased TAC (total antioxidant capacity)	*NFKB (p65), IL1B*, and *IL6* expression unchanged in white blood cells, IL-6 level unchanged in plasma. IL-1β increased after 24 h and decreased in 72 h in plasma	(164)
Simulated altitude (gas mixture of 11% O_2_, balance N_2_) for 30 min (group 1) and 60 min (group 2)	Group 1 – n=6 (age 33 ± 5.5 years; 2 females, 4 males) Group 2 – n=6 (age 26.5 ± 7.5 years; 3 females, 3 males)	Increased HIF-1α and VEGF levels in plasma of group 2	–	No significant changes were detected in circulating adhesion molecules (L-selectin, E-selectin, ICAM-1, and VCAM-1), Clara cell secretory protein (CC16), TNF-α, IL-6, and CRP	(165)
Simulated altitude (12%, 15%, 18%, and 21% O_2_ for 2 h in a normobaric hypoxia chamber)	n=20, males, age 23.1 ± 0.4 years		Exposure to 12% O_2_ promoted the increased urinary 8-isoprostane and plasma malondialdehyde levels and decreased plasma total antioxidant content and superoxide dismutase activity	Exposure to 12% O_2_ promoted the increased polymorphonuclear leucocytes chemotaxis and phagocytic activity, increased the expression of L-selectin, LFA-1 (lymphocyte function-associated antigen 1), Mac-1, FcγIIIBR, C1qRp, and C5aR on polymorphonuclear leucocytes, but did not alter plasma complement-C3a desArg/C4a desArg/C5a concentrations	(166)
Simulated altitude in an air-conditioned normobaric hypoxia chamber (equivalent to a height of 5,500 m, 2 h)	n=14, 12 males, 2 females, age 30 ± 6 years	Plasma HIF-1 and serum VEGF concentrations were not influenced by hypoxia, serum EPO increased. Peripheral blood cells synthesis increased HIF-1α protein in the cytosol whereas translocation into the nucleus was not increased	–	In peripheral blood cells, hypoxia caused a significant nuclear translocation of p50 NF-κB. Increased neutrophil phagocytic activity, but monocyte phagocytic activity was not changed. Decrease of TNF-α in monocytes and the decrease of IFN in CD4+ T-lymphocytes, no significant changes of IL-1β, IL-2, IL-4, IL-5, IL-10, and IL-17 either in monocytes or in T-lymphocytes	(167)
High-altitude (5,300 m, 5 days)	n=22, males, age 20–26 years	–	–	In subjects with AMS was revealed the decrease in *IL2, IL4, IL6ST, IL7, IL7R, IL10, IL17B, IL32*, *IL23R*, and *CCR7* expression and the increase in *IL13*, *IL17F,* and *CCL8* blood expression. Subjects with AMS were characterized by decreased IL-10 but increased IL-17F and CCL8 in plasma in comparison with those without AMS	(168)
High-altitude (3,200 m, 3 days)	n=102, males, age 30 ± 5.1 years	–	Plasma transferrin increased, whereas haptoglobin decreased. Transferrin of the AMS group significantly increased when compared with the non-AMS group, whereas haptoglobin of the AMS group was lower than non-AMS group	Concentrations of IL-1β, IL-6, and TNF-α increased, and these inflammatory cytokines in the AMS group were significantly higher than those in the non-AMS group. Plasma complement C3 increased after ascending to high altitude and had no significant difference between the AMS and non-AMS groups	(169)
High-altitude (3,800 m, 60 hours)	n=20, 13 males, 7 females, age 18–65 years	–	–	IL-1β, IL-6, IL-8, IL-10, TNF-α, and GM-CSF plasma concentrations increased. Increased IL-6 concentration was associated with higher AMS scores	(170)
Simulated altitude in a hypobaric chamber (4,875 m, 10 h)	n=20, 17 males, 3 females, age 25.5-31.8	–	AMS-resistant subjects had higher plasma HSP-70 compared with AMS-susceptible subjects	AMS-resistant subjects had higher plasma IL-1RA and adrenomedullin compared with AMS-susceptible subjects. Plasma macrophage inflammatory protein-1β was higher in AMS-susceptible than AMS-resistant subjects	(171)
Animal studies					
Albino outbred rats (11,500 m in decompression chamber, 1 month after)	Males	HIF-1α protein content in neocortex at normoxia was higher in hypoxia-susceptible animals	–	–	(133)
Wistar rats (11,500 m in decompression chamber, 1 month after)	n=60, males, 2–3 months old	*Hif1a* liver expression at normoxia was higher in hypoxia-susceptible animals	–	–	(142)
Wistar rats (11,500 m in decompression chamber, 1 month after)	n=50, males, 3–4 months old	Higher *Epas1* in non-activated macrophages of hypoxia-susceptible rats, higher *Hif1a* expression and HIF-1α level in LPS-activated macrophages of hypoxia-susceptible rats, lower *Vegf* expression and VEGF level in non-activated macrophages in susceptible rats	–	Higher *Il1b* and *Tnfa* expression in non-activated macrophages of susceptible rats, CCR7 level higher in LPS-activated macrophages of susceptible rats	(172)
C57Bl/6 mice (10,000 m in decompression chamber, 1 month after)	n=70, males, 1.5–2 months old	Colon *Hif1a* and *Hif3a* expression and serum HIF-1α content at normoxia were higher in hypoxia-susceptible animals	–	Colon *Nfkb1* and *Il10* expression were higher in hypoxia-susceptible animals	(173)
Sprague-Dawley rats (9,250 m in decompression chamber, immediately after three exposures)	n=115, males, 150 ± 15 g	Higher heart expression of HIF-1α and its dependent genes, including EPO, HO-1, and GLUT1, in tolerant animals, although VEGF expression was enhanced in the susceptible group	Hypoxia-susceptible animals demonstrated a three-fold increase in heart ROS levels and subsequent oxidative damage to proteins and lipids as compared with control. In tolerant animals, myocardial antioxidant enzymes, catalase, and superoxide dismutase were enhanced. Cytoprotective chaperones, HSP70 and HSP90, were elevated in hypoxia-tolerant animals	Hypoxia-susceptible animals demonstrated increased myocardial inflammation and infiltration, and greater CK-MB activity	(137)
Sprague–Dawley rats (9,754 m in decompression chamber, immediately after three exposures with 1-week interval)	Adult males, 150 ± 15 g	Tolerant animals demonstrated significantly higher plasma erythropoietin level as compared with susceptible ones	Tolerant animals demonstrated significant HSP70 upregulation in the cardiac tissue. The increased expression of transcription factor HSF-1 led to the downstream activation of other chaperones, including HSP90, HSP60, and HSP27. The higher induction of HSPs in tolerant animals contributed to higher NO synthesis mediated by both eNOS and iNOS activation	NF-κB expression in heart nuclear fraction increased only in susceptible rats. Increased in TNF-α expression in hypoxia-tolerant and hypoxia-susceptible animals, whereas it was more pronounced in susceptible ones	(140)
Sprague-Dawley rats (9,754 m in decompression chamber, immediately after three exposures with a 1-week interval)	Adult males, 150 ± 10 g	–	Tolerant animals demonstrated significantly lower plasma 8-isoprostane level and carbonyl proteins content. Transthyretin (TTR), glutathione peroxidase 3 (GPx-3), and paraoxonase 1 (PON1) were upregulated in tolerant animals, whereas apolipoprotein A-I (APOA1) was upregulated in susceptible animals. A higher level of haptoglobin and NO was observed in plasma of tolerant animals	–	(174)

### 3.4 Organism hypoxia tolerance, mitochondrial function, and NRF2

Energy metabolism is considered one of the main factors determining individual organism hypoxia tolerance. Approximately 90% of the energy required by the cell is provided by OXPHOS, which is significantly influenced by mitochondrial DNA (mtDNA) variations. Differences in mitochondrial functioning may largely be associated with baseline variations in the mitochondrial genome (149). To date, many studies have identified variations in mitochondrial gene expression and regulation associated with physiological response to changes in oxygen availability and oxidative stress (175). It is widely believed that mtDNA contributed to high altitude adaptation in innate high-altitude populations (176, 177). It was demonstrated that *eNOS*, *PPARA*, *HIF*, *ACE*, and *EPAS1* in the nuclear genome and *CYTB*, *ATP6*, *ND1*, *ND4*, and *ND5* in the mtDNA genome play an important role in Sherpa high-altitude adaptation (178). A connection between NRF2 and mitochondria was investigated—age-related decline in *Nfe2l2* expression, encoding NRF2, is connected with increased mtDNA damage in the cerebral cortex (179). It is possible that differences in mitochondrial gene expression, as well as NRF2 activity, may be observed in organisms depending on individual initial hypoxia tolerance; however, this question remains open.

Hypoxia susceptibility, including in different organism species, is largely determined by structural and functional mitochondrial features and oxidative stress levels (180–182). Differences in bioenergetic and redox systems in hypoxia-susceptible and hypoxia-tolerant animal species, including aquatic, high altitude, and subterranean, are already known (183). Genetic analysis of 12 hypoxia-tolerant mammal species demonstrated strong positive selection and convergent evolution of genes regulating ETC and OXPHOS (184). At the same time, the common adaptation mechanism in hypoxia-tolerant animals, including vertebrates and invertebrates, is due to the mitochondria’s ability to restore hypoxia-induced energy deficiency and reduce ROS production during reoxygenation (183). In species susceptible to hypoxia, such as most terrestrial mammals, hypoxia exposure significantly reduces mitochondrial OXPHOS and may lead to ATP deficiency, whereas anoxia leads to complete cessation of aerobic ATP production (13, 185, 186).

Ultrastructural and functional mitochondrial features, differences in respiratory chain enzymes’ kinetic properties, and central nervous system state and blood biochemical properties were revealed in animals with different hypoxia tolerance both within the same species and between different species. According to (180, 187), tolerant and susceptible organisms correspond to two fundamentally different “functional metabolic portraits”. Total oxygen consumption by hypoxia-tolerant rats under normoxic conditions is higher in comparison with susceptible rats. Taken together, all features of the hypoxia-susceptible animals’ “functional metabolic portrait” characterize them as a “risk” group (180).

In intact hypoxia-tolerant and hypoxia-susceptible animals, significant baseline structural morphological differences in cerebral cortex neuron mitochondria were revealed. Hypoxia-tolerant rats’ neurons were characterized by an increased (3.5-fold) number of small mitochondria in comparison with susceptible rats, reflecting greater OXPHOS intensity (135, 136, 180). Hypoxia-tolerant rats’ neuron mitochondria were also characterized by a greater number of cristae and high mitochondrial enzyme level (135, 136, 180). The number of cristae in mitochondria is a marker of their functional activity and reflects the distribution area of respiratory complexes and the intensity of OXPHOS occurring in them (188). The revealed hypoxia-tolerant rats’ mitochondrial features suggest higher baseline functional activity of the cerebral cortex neurons’ energy apparatus in comparison with susceptible rats, as well as higher mitochondrial enzyme activity, which may reflect initially greater ETC activity under normoxic conditions in tolerant animals in comparison with susceptible rats (135, 136, 180). Physiological and biochemical characteristics and differences between hypoxia-tolerant and hypoxia-susceptible organisms are quite fully characterized and are discussed in detail in the review (189).

Differences in mitochondrial enzyme activity in tissues of animals with different hypoxia tolerance, determining their aerobic energy metabolism features, correlate with many other aspects of their functional metabolic portrait, which are apparently largely genetically determined. According to the literature, hypoxia tolerance is connected with antioxidant defense enzyme activity, which is higher in people living in high-altitude regions due to constant oxygen deficiency exposure and ROS production (190). Interestingly, in women, mitochondria produce less ROS than men; this difference can also be observed in women’s activated antioxidant enzyme levels, largely due to estrogen effect (191). At the same time, female Wistar rats are more tolerant to hypoxia (192, 193). Probably, in hypoxia-tolerant animals, mitochondria also produce less ROS. After hypoxic exposure in the myocardium of hypoxia-tolerant Sprague-Dawley rats, the levels of enzymes such as superoxide dismutase and catalase, which protect cells from oxidative stress, were higher in comparison with hypoxia-susceptible animals (137, 140, 174). The activity of the most studied heat shock proteins (HSP60, HSP70, and HSP90), which stabilize membranes, in the myocardium of tolerant rats was significantly increased, indicating their ability to tolerate prolonged O_2_ deficiency exposure and more effective adaptation (137). Differences in adaptive capabilities and predisposition to inflammatory disease development in animals with different hypoxia tolerance are also confirmed by the fact that in susceptible Wistar rats after hypoxic exposure, the oxidative stress marker 8-isoprostane content increased, which was associated with the cell membrane damage and increased TGF-β levels (194). According to (137, 140) in hypoxia-susceptible rats’ myocardium, malondialdehyde content, which is formed under ROS action and is one of the oxidative stress markers, is almost eight times higher.

Low organism hypoxia tolerance may be linked to inefficient metabolism, such as an initially greater dependence on glycolysis rather than OXPHOS, or mitochondrial dysfunction. Such inefficient metabolism itself may cause a proinflammatory background through ROS production, mtDNA, low ATP production, etc. For instance, throughout aging, mitochondrial biogenesis and antioxidant system functioning are disrupted, accompanied by the increased oxidative stress and inflammation (195). It was established that when quality control mechanisms are disrupted, damaged mitochondria cannot be effectively removed, leading to increased mitochondrial membrane permeability and, consequently, mtDNA release into the cytoplasm. Released mtDNA acts as a DAMP, activating innate immune responses through pathways such as cGAS-STING, NLRP3 inflammasome, and TLR9. This leads to inflammatory mediators (e.g., IL-1β) and ROS formation. These signaling molecules not only exacerbate mitochondrial membrane damage and mtDNA release but also further impair quality control by suppressing autophagy or disrupting mitochondrial biogenesis. This creates a vicious cycle from mitochondrial quality control imbalance and mtDNA release to inflammation and oxidative stress, leading to further quality control deterioration (196). However, mtDNA level assessment in organisms with different hypoxia tolerance was not performed.

High HIF-1 content both in humans susceptible to HAPE before exposure (197) and in hypoxia-susceptible rats (133, 142, 173) may be associated with decreased mitochondrial mass and lower OXPHOS efficiency (12). This may also be associated with mtDNA mutations; however, further study is required. Greater glycolysis utilization in susceptible organisms leads to background acidosis and inflammation. This in turn may contribute to higher rates of tumor initiation and progression against the background of chronic inflammation, as demonstrated in the colorectal cancer model (173, 198).

Differences in mitochondrial functioning (135, 136, 180, 187) were detected 1 month after the hypoxic exposure; therefore, they may not be the cause of the initial low tolerance but a consequence of hypoxia exposure and/or the result of long-term adaptation to it. A study of mitochondria in organisms with different hypoxia tolerance before its determination in the decompression chamber is necessary. This may be either by obtaining skeletal muscle biopsies (199) with subsequent mitochondria study in them and determination of animal hypoxia tolerance, or by development of highly reproducible methods for hypoxia tolerance determination without oxygen deficiency exposure.

Thus, susceptible organisms exhibit signs of mitochondrial dysfunction, which may be both a cause and a consequence of low hypoxia tolerance. Regardless of the causal connection, dysfunctional mitochondria are a source of both ROS and DAMPs, which may contribute to inflammation. Furthermore, basally high HIF-1α activity in susceptible organisms may promote NF-κB activation. These mechanisms may underlie the proinflammatory phenotype in susceptible organisms, which will be discussed further.

### 3.5 Organism hypoxia tolerance and the proinflammatory phenotype

Bai et al. (152) revealed differences in adaptive and immune responses in high-altitude residents. A total of 95 representatives of the Han living at moderate altitudes (1,507 m), 95 Tibetans, and 98 representatives of the Han living at high altitudes (2,900–4,287 m) were studied. Hematocrit (HCT), mean erythrocyte volume (MCV), and mean erythrocyte hemoglobin content (MCH) values, CD19+ B lymphocyte count, Erb-B2 receptor tyrosine kinase 3 (ErbB3) and tumor necrosis factor receptor II (TNF-RII) levels, and serum HIF-1α, HIF-2α, and PHD2 levels were decreased, whereas the frequency of SNPs in *EPAS1* and *EGLN1* genes was increased in Tibetans living at high altitudes in comparison with Han living at high altitudes. Furthermore, in comparison with people living at moderate altitudes, people living at high altitudes had lower hemoglobin concentration, hematocrit, MCV, and MCH, higher mean erythrocyte hemoglobin concentration (MCHC), fewer CD19+ B cells and CD4+ T cells number, lower CD4/CD8 ratio, more CD8+ T cells and CD16/56 NK cells, decreased MIP-1b and IL-8 levels, increased thrombomodulin levels, decreased levels of hypoxia-related factors including HIF-1α, HIF-2α, and PHD2, and higher frequency of *EGLN1* rs2275279 polymorphism (152). The obtained results indicate that in Tibetan populations, adaptation to high-altitude hypoxia could be mediated by changes in immune cell functioning, cytokine levels, and hypoxia response regulating gene expression during evolution. The authors suggest that genetic polymorphisms of hypoxia response regulating factors may determine differences in immune system functioning in different high-altitude populations. However, inflammatory regulation represents only one of several mechanisms contributing to hypoxia tolerance, which also involve metabolic, cardiovascular, respiratory, and cellular adaptive responses.

Recently, it was established that *EPAS1*, *EGLN1* (egl-9 family hypoxia-inducible factor 1), eNOS (endothelial nitric oxide synthase), ACE (angiotensin-converting enzyme), and TIMP3 (tissue inhibitor of metalloproteinase 3) genes are significantly associated with HAPE susceptibility in the Japanese population (200). In addition, in innate populations of both Andean and Tibetan high-altitude populations, signs of evolutionary selection of genes regulating inflammatory responses *IL1A*, *IL1B*, *IL6*, *NOS1*, *NOS*2, and *TNF* were revealed (201, 202). High *IL6* expression is connected with the high-altitude disease development, as it plays an important role in adaptation to hypoxia. Under oxygen deficiency, IL-6 promotes hematopoietic stem cell proliferation, and its effect on erythropoiesis is synergistic with other proinflammatory cytokines, including IL-1α and TNF-α (203). It was demonstrated that rs1800796 and rs1524107 *IL6* are associated with high HAPE risk (204). Probably, a low *IL6* expression level may prevent hypoxia-induced chronic low-grade systemic inflammation. Another important gene undergoing selection in both Tibetan and Andean groups is *TNF* (202), a multifunctional proinflammatory cytokine. Increased TNF-α levels were detected in people making short ascents to high-altitude regions (170). The adaptive *TNF* variant is unknown, but a low *TNF* expression in response to chronic hypoxia may be important for preventing acute inflammation at high altitude (205). It is also known that highlanders (2,800 m) have lower blood CD3+, CD4+, and CD20+ lymphocytes content in comparison with plain residents (930 m), which may indicate low proinflammatory potential of the former (206).

Given the role of HIF in regulating many genes responsible for various processes in the organism and its well-established connection with NF-κB and NRF2, higher baseline *Hif1a* expression in hypoxia-susceptible organisms may influence the predisposition to the development and severity of some inflammatory and tumor diseases (142, 173, 198, 207, 208). Probably, this is largely due to different oxidative stress levels and antioxidant defense enzyme activity. Thus, it was demonstrated that during systemic inflammatory response induced by LPS, higher *Hif1a* gene expression in hypoxia-susceptible animals is accompanied by the increased *Nfkb* expression and more severe disease course (142). There are data that COVID-19 severity depends on organism hypoxia tolerance (209).

Polarization of macrophages—innate immune cells—can be regulated by energy metabolism, including that changing under hypoxia and ischemia influence (210). The key role of mitochondria in macrophage functioning was characterized (211). Changes in metabolism and mitochondrial physiology may underlie macrophage polarization and activation. These changes include alterations in oxidative metabolism, mitochondrial membrane potential, and the tricarboxylic acid (TCA) cycle, as well as mitochondrial ROS and mtDNA release and mitochondrial ultrastructure transformation. The role of succinate in macrophage proinflammatory phenotype formation was revealed and described (119, 212). ROS production caused by succinate oxidation contributes to this phenotype development (213). Previously, we demonstrated that, in comparison with hypoxia-tolerant rats, unactivated macrophages derived from blood monocytes of hypoxia-susceptible Wistar rats exhibit lower VEGF gene and protein expression but higher expression of proinflammatory cytokine *Il1b* and *Tnfa* genes, as well as the *Epas1 (Hif2a)* gene (**Table 1**). Upon LPS activation, macrophages from Wistar rats susceptible to hypoxia respond by increasing the *Hif1a* expression and HIF-1α protein, HGF, and chemokine receptor CCR7 content, indicating proinflammatory polarization (172). Apparently, immune cells of hypoxia-susceptible animals demonstrate functional activation signs comparable with mild but long-term maintained hypoxia conditions. Macrophages of hypoxia-susceptible animals are characterized by enhanced reactivity to LPS stimulation and more pronounced HIF-1α activation, increased *Tnfa*, *Il1b*, and *Nos2* expression, and increased CCR7 and HGF content. This allows to consider low hypoxia tolerance as a phenotype with a chronic proinflammatory background, which predetermines the organism’s response to subsequent pathological influences and creates conditions for accelerated initiation of inflammatory and tumor processes.

Thus, there are differences in the basal activity of HIF, antioxidant system, and inflammation regulation that are associated with the initial individual organism hypoxia tolerance and the proinflammatory phenotype. The mechanisms underlying the proinflammatory phenotype in hypoxia-susceptible organisms remain to be elucidated. Additional molecular-genetic, transcriptomic, and other studies are required to determine the mechanisms of the proinflammatory phenotype and aim at identifying such organisms, since they constitute the largest risk group for the development of inflammatory and tumor diseases, as well as those associated with altitude and hypoxia exposure. Probably, the proinflammatory phenotype and low hypoxia tolerance in such organisms are genetically determined, as well as epigenetically through regulation of mitochondrial functioning, which leads to constitutively high levels of DAMPs, oxidative stress, and succinate.

## 4 Systemic hypoxic exposure as a model for studying the hypoxia tolerance and proinflammatory phenotype connection

If the proinflammatory phenotype in hypoxia-susceptible organisms is indeed driven by constitutively elevated HIF activity, mitochondrial dysfunction, and enhanced DAMP signaling, then one would expect that exposure to systemic hypoxia—such as during the high-altitude ascent—would amplify these differences and reveal their functional consequences. The following section reviews available evidence on how HIF, NF-κB, and NRF2 responses change during high-altitude exposure, with particular attention to studies that have stratified individuals by AMS susceptibility. High-altitude mountaineering involves exposure to a hypobaric hypoxic environment, which leads to a number of physiological, immunological, and metabolic changes that remain poorly understood. As mentioned above, HIF-1α regulates both innate and adaptive immune cell functioning and metabolism (214, 215). Given the connection between HIF and NF-κB signaling pathways, it can be assumed that hypoxia caused by high-altitude stay may lead to the development of high-altitude diseases and systemic inflammatory response. The connection between hypoxia and inflammation is widely discussed in studies conducted under high-altitude conditions (170). On the one hand, high-altitude hypoxia leads to HIF signaling pathway activation. On the other hand, it promotes NF-κB translocation to the nucleus through IKKβ pathway activation, which additionally enhances inflammatory gene transcription (205).

### 4.1 Hypoxia-induced inflammatory and immune responses during high-altitude ascent

A number of previous studies presented evidence that during the first few days of acclimatization, climbers are characterized by increased levels of some inflammatory mediators in peripheral blood (94, 115, 160–167, 170) and that acute hypoxic exposure affects immune cell functioning (216–218). Short-term (up to 4 days) stay of animals and humans under hypoxia (at altitudes above 3,400 m) led to increased levels of serum inflammation markers—IL-6 and C-reactive protein (CRP) (160, 161).

According to peripheral blood RNA sequencing during 3 days of high-altitude acclimatization in healthy sea level volunteers, on the first day of high-altitude stay (3,800 m), an increased expression of inflammation-related genes was observed, including a significant increase in *HMGB1* (high-mobility group box 1)—a DAMP that enhances immune response during tissue damage, and another DAMP—*S100A8* as well as, *TLR4*, *LY96*, encoding lipopolysaccharide-binding protein (MD-2), and *IL18*. These results indicate that ascent to altitude activates inflammatory signaling pathways (162).

A recent multi-omics study of blood samples from mountaineers during gradual ascent to a 7,546-m altitude, including transcriptomic analysis of 375,722 immune cells, as well as plasma metabolome and lipidome, was conducted (163). The results (**Table 1**) indicate that circulating immune cell composition in mountaineers changes significantly during high-altitude ascent. On days 11 and 16, the proinflammatory potential of monocytes and dendritic cells significantly increased, with increased *IL1B*, *TNF*, and *NF-κB1* expression in them. The authors noted that hypoxia and *HIF1A* activation may be key factors in regulating and activating inflammatory responses during altitude ascent. *HIF1A* expression levels and dependent genes regulating glycolysis and hypoxia response significantly increased; moreover, increased expression of antioxidant-related genes—*SOD2*, *PRDX4*, and *TXN*, including the gene *NFE2L2* encoding NRF2, was also revealed. According to plasma analysis, enhanced glutamine and fatty acid metabolism was revealed.

Malacrida et al. (164) studied the dynamics of mRNA levels of key transcription factors HIF-1α, HIF-2α, NF-κB, and NRF2, as well as proinflammatory cytokines and oxidative stress markers in white blood cells of 15 mountaineers before and after rapid passive ascent to a 3,830-m altitude (after 24 and 72 h). Hypoxia temporarily increased the *HIF1A* mRNA levels in white blood cells over time, peaking at 24 h. While mRNA levels encoding HIF-2α and NRF2 increased over time, NF-κB, IL-1β, and IL-6 mRNA levels remained unchanged, which was likely associated with lower altitude and study duration in comparison with data in the work (163). Plasma IL-6 levels did not change; the IL-1β level increased at 24 h and decreased at 72 h. In dynamics, the ROS formation rate and level of oxidative stress-induced damage markers significantly increased. Transcription factor gene expression analysis suggests that HIF-1α is the leading transcription factor in the organism’s response to subacute hypoxic exposure. Prolonged hypoxic exposure led to a switch from HIF-1α to HIF-2α/NRF2 signaling pathways. These results are important for understanding the temporal regulation of transcription factors, inflammatory response, and ROS homeostasis involved in the reaction to hypoxia and the development of oxygen deficiency-related diseases. However, these studies (162–164) included 15, 11, and 15 healthy volunteers of both sexes, i.e., the sample sizes were insufficient; moreover, age, sex differences, and initial organism hypoxia tolerance were not considered. Further studies of the three key transcription factors (HIF, NF-κB, and NRF2) in organisms with different hypoxia tolerance both under normoxia and under oxygen deficiency are needed.

Proinflammatory response activation under oxygen deficiency exposure was also demonstrated under normobaric hypoxia (**Table 1**). In healthy volunteers subjected to hypoxia *in vivo*, neutrophil chemotaxis, phagocytic activity, and ROS production were enhanced (166). Furthermore, under hypoxia, HIF-1α and NF-κB content, as well as neutrophil phagocytic activity, excluding that of monocytes, increased in peripheral blood cells of healthy volunteers (167). However, this work included both men and women. Later, in an *in vitro* study, it was demonstrated that 24 h normobaric hypoxia (0.5%-3% O_2_) exposure did not affect neutrophil phagocytic activity (219). At the same time, increased monocyte phagocytic activity under hypoxia was demonstrated by many researchers (220, 221). It was also investigated that hypoxia affected myeloid-derived suppressor cells in mice (222). Nevertheless, these works did not consider individual organism hypoxia tolerance. We revealed differences in monocyte phagocytic activity in hypoxia-tolerant and hypoxia-susceptible Wistar rats—after *in vitro* and *in vivo* hypoxic exposure, monocyte phagocytic activity was higher in tolerant animals in comparison with susceptible rats (223).

Notably, as a rule, no special stratification according to hypoxia tolerance before ascent was performed in high-altitude studies. Given that hypoxia-susceptible individuals even at the sea level exhibit elevated HIF-1α and proinflammatory cytokine production (as discussed in Section 3), it is plausible that the exaggerated inflammatory responses observed in AMS-susceptible mountaineers represent not merely a reaction to altitude but an amplification of their preexisting proinflammatory phenotype.

### 4.2 Differential inflammatory responses in individuals with and without high-altitude diseases

A separate line of investigation directly compared individuals who develop high-altitude illnesses with those who remain tolerant. These comparative studies provide critical insights into whether the inflammatory response is a cause or a consequence of altitude-related pathology, and, importantly, whether preexisting differences in immune status can predict AMS susceptibility. Acute increase in proinflammatory cytokines such as IL-6, IL-8, and TNF-α during mountain ascent in some people may contribute to the development or be a consequence of high-altitude diseases such as AMS, HAPE, and HACE (224, 225). Inflammatory mediator level at altitude differs depending on whether the patient develops AMS or not. At the same time, in AMS-tolerant individuals, the inflammatory response under high-altitude conditions was accompanied by anti-inflammatory mechanism activation, which allows avoiding tissue damage, whereas in AMS-predisposed individuals, immune response dysregulation with excessive proinflammatory pathway activation and insufficient anti-inflammatory molecule compensation were observed (168, 226). For instance, individuals with AMS were characterized by decreased IL-10 levels but increased IL-1β, IL-6, and TNF-α levels in blood plasma in comparison with individuals without AMS (168, 169, 226). At sea level, these parameters did not differ (169). In studies (227, 228), alveolar lavage fluid from mountaineers diagnosed with HAPE in some but not all cases showed increased neutrophil content, proinflammatory cytokine level, and chemotactic factor level. This indicates that inflammation may be a causal factor in HAPE, leading to greater microvascular vessel permeability in the lungs. However, not in all HAPE cases were signs of inflammation detected (228), indicating that in humans, the development of inflammatory reactions in the lungs may be a consequence of alveolar capillary barrier disruption and edema. Thus, although it is suggested that hypoxia caused by high-altitude exposure may contribute to acute increases in some proinflammatory mediators under certain ascent profiles, it is unknown whether this systemic inflammatory response is a consequence or a contributing factor to the development of high-altitude diseases such as AMS, HAPE, and HACE (12, 225).

High expression of proinflammatory *IL1B* was reported to correlate with the AMS symptoms worsening (229). Julian et al. (2011) (171) demonstrated (**Table 1**) that after 4 and 9 h of a simulated 4,875-m “altitude” in a decompression chamber, AMS-tolerant individuals had higher blood levels of IL-1RA—interleukin-1 receptor agonist. After 4 h of hypoxia exposure, the MIP-1 (macrophage inflammatory protein-1) level was higher in AMS-susceptible individuals in comparison with tolerant ones. Biomarkers such as IL-6, IL-8, IL-10, VEGF, TNF-α, MCP-1 (monocyte chemoattractant protein-1), and matrix metalloproteinase MMP9 did not differ in peripheral blood between AMS-tolerant and susceptible individuals (171).

### 4.3 Possible biomarkers of AMS susceptibility

Nevertheless, the question of whether the inflammatory response at altitude is driven by preexisting differences in immune status remains unsolved. One approach to addressing this question is to examine whether molecules measured at sea level can predict AMS susceptibility before ascent, thereby distinguishing preexisting traits from altitude-induced changes.

It was demonstrated that people who differ according to the AMS sensitivity during altitude ascent vary in the levels of certain molecules at sea level, prior to ascent. Thus, AMS-susceptible people are characterized by a proinflammatory phenotype already at sea level. Levels of molecules such as IGFBP6 (insulin-like growth factor binding protein 6), Dkk4 (Dickkopf WNT signaling pathway inhibitor 4), serum amyloid A1 (SAA1), and IL-17RA (interleukin-17 receptor A) in blood under normoxia differ between AMS-tolerant and AMS-susceptible people. In AMS-tolerant people, IGFBP6 levels were higher in blood, but the Dkk4, SAA1, and IL-17RA levels were lower. These parameters may be used to predict AMS development before altitude ascent (139). After 9 h of hypobaric hypoxia, the abundance of proteins related to TCA cycle and glycolysis were significantly reduced in AMS-tolerant people, but not in those susceptible (143). The authors suggested that AMS-tolerant individuals could reduce oxygen consumption by repressing TCA cycle and glycolysis and reduce energy consumption by decreasing protein degradation and synthesis compared with susceptible ones. The inflammatory response might be decreased resulting from the repressed TCA cycle (143). Recently, it was demonstrated that serum IL-2, IL-17A, and TNF-α levels assessed before ascent can be used as potential biomarkers of AMS susceptibility at 3,700- and 5,000-m altitudes (230). Possible biomarkers for hypoxia tolerance determination are discussed in detail in the recently published review (231). As of today, no biomarkers for AMS have entered clinical routine and AMS diagnosis is based primarily on self-reported symptoms (10).

### 4.4 *Ex vivo* approaches for the investigation of organism hypoxia tolerance

*Ex vivo* blood cell incubation and activation is a promising approach for studying immune responses and reproduces conditions closest to *in vivo* conditions. For instance, *ex vivo* LPS stimulation of blood cells leads to IL-1β production (232–234). The cytokine level in serum or plasma reflects the immune system functional state, whereas in situations associated with deficiency or imbalance of regulatory factors, it is necessary to assess the ability of blood cells to secrete cytokines. It was established that spontaneous production of the proinflammatory cytokines IL-6 and TNF-α, as well as the anti-inflammatory cytokine IL-10, during 24-h blood incubation under normoxia was higher in hypoxia-susceptible animals before hypoxia tolerance determination in the decompression chamber. Increased production of these cytokines in susceptible rats may be due to their proinflammatory phenotype. It is consistent with data that in susceptible Wistar rats unactivated macrophages are characterized by increased *Tnfa* and *Il1b* expression in comparison with tolerant animals (172). Furthermore, before exposure in the decompression chamber, *ex vivo* stimulation of blood cells with a mitogen complex (LPS, phytohemagglutinin—PHA, and concanavalin A—ConA) promoted the increased IL-1β production only in susceptible rats in comparison with spontaneous production (235). Thus, for searching biomarkers of high sensitivity to AMS and development of other high-altitude pathologies at sea level, the use of *ex vivo* blood cell incubation under various conditions appears promising, as it allows to avoid the exposure to the organism and identifying the risk group.

According to the literature, elevated *ex vivo* LPS-stimulated blood TNF-α and IL-1β levels are associated with high plasma CRP levels in healthy volunteers and indicate a proinflammatory phenotype (236). This systemic proinflammatory phenotype may contribute to the low-grade inflammation, as well as reflect the presence of inflammation in vessels or other organs and tissues. Both in studies of healthy volunteers before mountain ascent and in intact animals before hypoxic exposure in the decompression chamber, groups with a proinflammatory phenotype with initially high levels of proinflammatory cytokine production by blood cells were identified. This proinflammatory phenotype is connected with low hypoxia tolerance determined after hypoxic exposure in mountains or decompression chamber. It is now commonly considered that mechanisms of hypoxia, inflammation, and immune responses are closely connected; therefore, differences in initial organism hypoxia tolerance may influence the course of inflammatory processes, including during altitude ascent. However, initial organism hypoxia tolerance is rarely taken into account in studies. Further research is needed to identify markers of proinflammatory phenotype and low individual hypoxia tolerance without the hypoxic exposure to the organism.

Furthermore, it remains unrevealed whether this proinflammatory phenotype is genetically determined or is shaped by external factors. One possible explanation is offered by the concept of trained immunity—long-term functional reprogramming of innate immune cells.

## 5 Trained immunity as a mechanistic link between the low hypoxia tolerance and the proinflammatory phenotype

Organism hypoxia tolerance, as discussed above, is determined by genetic, epigenetic, and phenotypic factors and largely determines the development of the inflammatory response during altitude ascent. The initial hypoxic status, higher HIF-1α activity, and metabolic and mitochondrial features in hypoxia-susceptible organisms may determine not only their proinflammatory phenotype but also features of trained immunity development—innate immune cell memory.

### 5.1 HIF and hypoxia in the immune cell function

As discussed above, HIF activation in immune cells may determine the severity of various diseases. In recent years, it was demonstrated that oxygen deficiency and HIF activation through metabolic status changes play a key role in innate and adaptive immune cell functioning (237–239). Thus, HIF connects metabolic and immune/inflammatory processes. After HIF activation, innate immunity gene expression containing HREs in promoters increases. They reach maximum expression upon NF-κB activation after direct contact with the pathogen, which additionally enhances the HIF transcription. Thus, HIF is an important regulator of both innate and adaptive immune cells (**Figure 2**) and inflammatory processes (240–267). In general, many other factors, for instance, cytokines and chemokines, combine with hypoxia, allowing control of immune cell development and their functions; therefore, the final hypoxia effects depend on which a co-stimulus is present.

**Figure 2:**
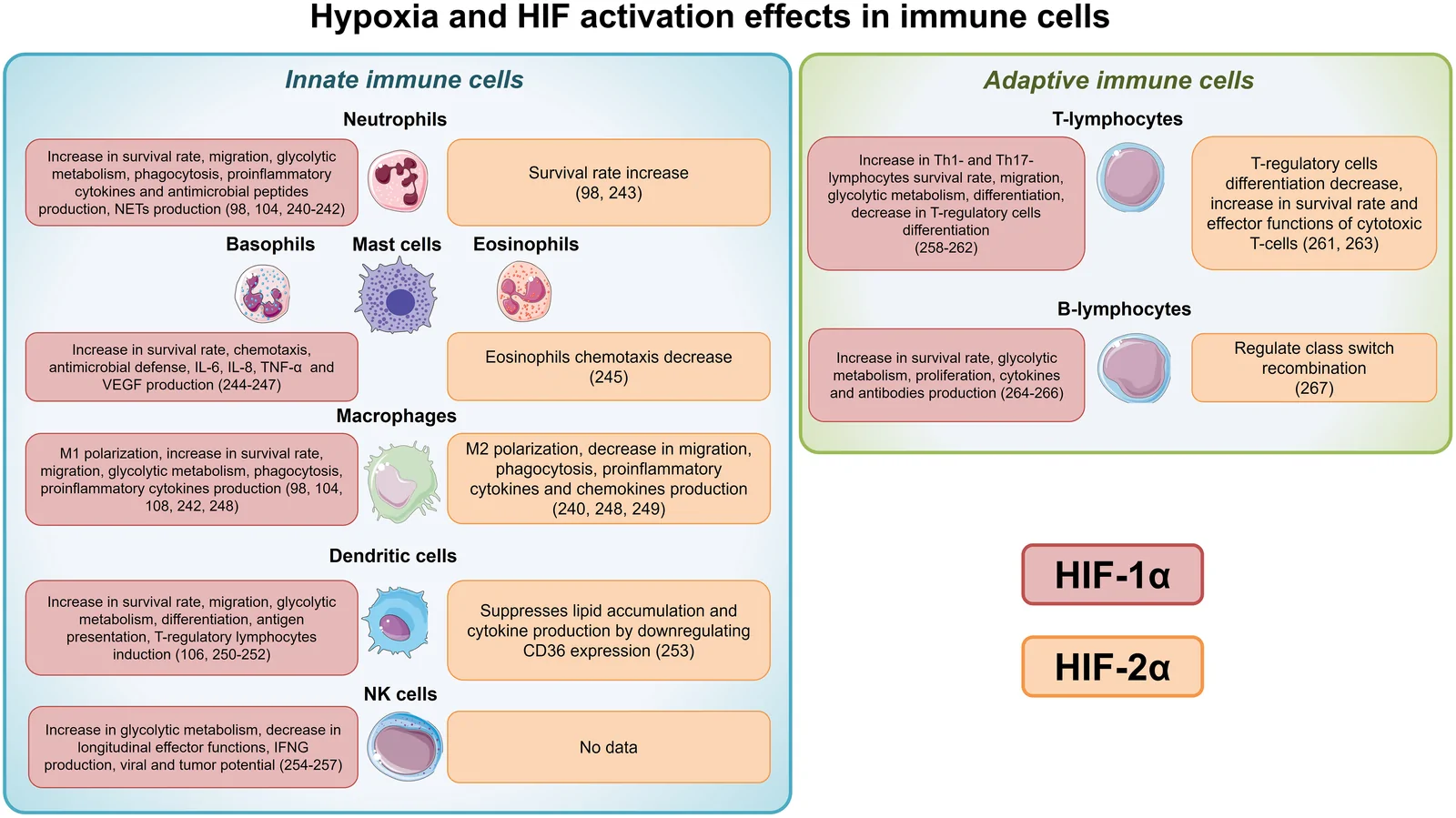
Hypoxia and HIF isoforms (HIF-1α and HIF-2α) activation effects in innate (neutrophils, basophils, eosinophils, mast cells, macrophages, dendritic cells, and NK cells) and adaptive (T-lymphocytes and B-lymphocytes) immune cells. HIF activation predominantly promotes a proinflammatory functional profile in most innate and adaptive immune cell subsets. More functions currently were investigated for HIF-1α. The red fill color corresponds to the activation of HIF-1α, the orange fill color to HIF-2α.

### 5.2 Trained immunity mechanisms

Trained immunity is defined as a long-term functional reprogramming of innate immune cells, triggered by the contact with PAMPs and/or DAMPs. This reprogramming is mediated by sustained epigenetic modifications such as histone methylation and acetylation affecting DNA accessibility for transcription factors, and metabolic shifts. This results in altered responses upon the subsequent stimulation, compared with untrained cells (23–25, 268). In contrast to untrained immune cells, trained cells are capable of exhibiting more pronounced responses upon repeated exposure to various stimuli. These qualitative changes in immune cells functioning are associated with differential regulation of expression of a number of proinflammatory genes, such as *IL1B*, *IL6*, and *TNFA*, as well as anti-inflammatory *IL10*, due to changes in chromatin remodeling, histone modifications, and cellular metabolism reprogramming (23, 24). In particular, the increased IL-1β, IL-6, and TNF-α production and the decreased IL-10 production indicate the development of a proinflammatory type of trained immunity, whereas increased IL-10 production indicates the anti-inflammatory type (24). Differences in trained immunity development under hypoxic exposure in hypoxia-tolerant and hypoxia-susceptible organisms may determine the risk of development and severity of inflammatory and tumor diseases.

It is considered that innate immune cell memory can persist from weeks to months and, in the case of live vaccines—up to 5 years (23, 269). Initially, trained immunity was identified in mature myeloid cells, but their short lifespan (several days) raised doubts about how their effect could last months or years. This paradox was resolved by the discovery that trained immunity can also be formed at the level of long-lived hematopoietic stem cells in the bone marrow (“central trained immunity”), not only in mature myeloid cells (“peripheral trained immunity”). This leads to sustained development of myeloid cells with a proinflammatory phenotype and may contribute to the development and maintenance of chronic inflammatory conditions, thereby playing a key pathogenetic role in a number of chronic inflammatory diseases (270). Early studies emphasized the protective role of trained immunity against a number of pathogens, but then it became evident that excessive trained immunity activation may also contribute to chronic inflammatory processes and development of many diseases, which was discussed in the maladaptive trained immunity concept (271–274). This phenomenon was termed maladaptive trained immunity, referring to a persistent state of memory imprinted in innate immune cells that, instead of eliciting protective responses, results in detrimental effects such as sustained inflammation and immune dysregulation. While short-term or controlled exposures, such as vaccination or acute infection, may induce adaptive trained immunity that enhances organism protection, chronic or repeated stimuli may cause the maladaptive immunity, sustaining inflammation and contributing to disease development.

It is known that in response to PAMP and DAMP binding to pattern recognition receptors (PRRs), NF-κB is activated, inducing proinflammatory and anti-inflammatory cytokine production, such as IL-1β and IL-10 (275, 276). Both PAMPs such as β-glucan, BCG, and LPS and DAMPs such as iron-containing tetrapyrrole heme, intermediate filament vimentin, oxidized low-density lipoproteins (oxLDL), and mtDNA can lead to epigenetic changes in innate immune cells and trained immunity formation (23, 25). DAMP-induced trained immunity is less studied in comparison with PAMP-induced trained immunity (277). It is important to note that trained immunity represents a long-term adaptation of innate immune cells, not a specific transcriptional or functional program: different stimuli, such as β-glucan, LPS, or BCG, can induce differing in severity and direction trained immunity programs (23). The predominant cell population providing PAMP and DAMP recognition and trained immunity response implementation are monocytes and macrophages expressing PRRs (23). Macrophages are a heterogeneous and highly flexible innate immune cell group capable of changing under the stimuli secreted by microenvironment cell, PAMP, and DAMP influence. One of the main functions of these cells is phagocytosis, as well as synthesis and production of various cytokines (278). As it was already stated, the established differences in macrophage functioning in hypoxia-tolerant and hypoxia-susceptible animals (172) may be associated with features of trained immunity formation in response to endogenous DAMP stimulation.

### 5.3 Hypoxia-induced trained immunity

Epigenome modification, which underlies trained immunity, is closely connected with cellular metabolism, since metabolites can modify chromatin and alter epigenetic factor activity (279–281). One of the mechanisms of trained immunity development is HIF-1α-dependent aerobic glycolysis activation and OXPHOS decrease (282, 283). This increase in glycolysis was associated with changes in the epigenome, namely, the increased deposition of the transcriptional permissive mark H3K4me3 at regions associated with the promoters of glycolytic enzymes (281, 282). Increased mTOR- and HIF-1α-mediated aerobic glycolysis was revealed in both BCG- and β-glucan-stimulated monocytes (282–284). HIF-1α stabilization is crucial for the trained immunity induction, promoting IL-1β induction, glycolysis, and histone demethylase expression changes (119, 281, 282). Furthermore, the important role of lactate as an energy source in trained immunity development was investigated (285). Metabolic changes, in particular fumarate accumulation, led to HIF activation, promoting proinflammatory gene expression, as well as monocyte epigenetic reprogramming induction through KDM5 histone demethylase inhibition. As a result, permissive histone marks (H3K4me3) accumulate in proinflammatory gene promoters in monocytes, i.e., these regions are open for binding to corresponding transcription factors (286, 287).

Thus, trained immunity development depends, among other things, on hypoxia and HIF-1 activation; however, further studies on these process mechanisms are needed. Most of the published literature linking hypoxia to trained immunity is based on *in vitro* or *in vivo* studies where it is difficult to determine the true degree of hypoxia experienced by innate immune cells. Data on the role of hypoxia, activated signaling pathways in trained immunity stimulation were obtained (282), which found that mice with knockout of the gene encoding HIF-1α in myeloid cells lacked β-glucan-induced trained immunity and more often died after subsequent *S. aureus* exposure. AKT/mTOR signaling pathway activation upon β-glucan exposure enhanced *HIF1A* gene expression and HIF-1α protein activity and also altered cellular metabolism, stimulating glycolysis. This was associated with histone modifications of H3K4me3- and H3K27ac, activating innate immunity genes, although the exact causal connection was not established (282). Probably, the activity of enzymes that perform histone modifications may differ depending on the individual organism hypoxia tolerance; however, such data are not presented in the literature.

Since systemic hypoxia determines the organism’s proinflammatory background, and HIF regulates innate immune cells, it is likely that high-altitude and plain residents, as well as hypoxia-tolerant and hypoxia-susceptible organisms, could differ in trained immunity state in response to hypoxic exposure. As it was already mentioned, during high-altitude acclimatization in healthy volunteers, increased expression of several inflammation related genes was observed, including a significant increase in DAMPs—*HMGB1* and *S100A8* (162). HMGB1, depending on dose and context, activates RAGE, leading either to AMPK/SIRT1-mediated tolerance or to mTOR- and HIF-1-induced trained phenotype with increased proinflammatory cytokine expression and glycolysis activation (288). Upon interaction with PRR TLR2 and TLR4, HMGB1 induces the NF-κB-dependent transcription of proinflammatory and anti-inflammatory cytokine genes (289). Probably, in hypoxia-susceptible organisms, DAMPs are pronouncedly produced during the hypoxic exposure, including HMGB1 and S100A8, which contribute to trained immunity formation with increased proinflammatory cytokine expression, whereas in tolerant organisms, such a response is not observed.

DAMPs can be formed in response to cell damage, which inevitably occurs during the sublethal hypoxic exposure in the decompression chamber at a critical “altitude”. Hypoxia tolerance determination or mountain ascent involves hypoxic exposure of varying severity to the organism. It was demonstrated that the decompression chamber exposure leads to increased proinflammatory IL-1β production by blood cells, but decreased IL-10 production in hypoxia-susceptible Wistar rats (290). This indicates that besides a preexisting proinflammatory phenotype in hypoxia-susceptible animals, detected both before and after the sublethal hypoxic load, hypoxia provides the immunomodulatory effect on cytokine production by blood cells, which persists 1 month after the exposure. This confirms the data on the influence of oxygen deficiency on immune cells’ functional capacity (215). Our data indirectly confirm suggestions (282, 291) that hypoxia can activate trained immunity. At the same time, if hypoxic exposure indeed has such an effect, then organism hypoxic training or hypoxic therapy becomes even more relevant. In this case, initial organism hypoxia tolerance should be considered, since in hypoxia-susceptible organisms, hypoxic training may lead to a proinflammatory immune cell phenotype and a more severe course of subsequent diseases.

In addition to trained immunity, tolerance is also discussed, being associated with predominant use of OXPHOS by immune cells and decreased proinflammatory cytokine production (288), which reduces reactivity to subsequent exposures. It is suggested that any PAMP or DAMP can lead to immune cell training or tolerance depending on exposure conditions; low doses or acute exposures cause the increased reactive state, whereas high doses or chronic exposures cause a refractory state (288). Tolerant innate immune cells produce more IL-10 and less IL-12, potentially shifting T-cell polarization toward regulatory or Th2 (288). It was demonstrated that PRR thresholds are species-specific (e.g., TLR4/LPS sensitivity, Dectin 1 isoforms, and inflammasome activation), so the same DAMP can bias toward training versus tolerance across species (288, 292). Possibly, the balance of trained immunity and immune tolerance depends on the initial organism hypoxia tolerance. Nevertheless, these issues were not described in the literature, and studies on trained immunity and tolerance are generally conducted *in vitro* and do not consider initial organism hypoxia tolerance. Probably, during the sublethal hypoxia exposure in the decompression chamber, formed DAMPs contribute to innate immune cell memory development; at the same time, in hypoxia-tolerant animals, due to the tolerance strategy, the response to a repeated stimulus is more effective, whereas in hypoxia-susceptible animals, it has a maladaptive character with the development of immune disorders and inflammation. Nevertheless, the concept of trained immunity was established recently, and further studies are needed to elucidate its mechanisms. Similarly, in humans during mountain ascent, trained immunity and adaptation (e.g., increased performance in athletes) may be developed, as well as maladaptation and proinflammatory response activation with development of high-altitude diseases.

### 5.4 Mitochondria-induced trained immunity

It remains unclear why some DAMPs induce the sustained innate immune memory whereas others do not. Many damage-associated DAMPs (e.g., ATP and nuclear histones) do not induce long-term trained immune responses—some instead lead to transient activation or tolerance. It is necessary to identify which stimulus features (molecular structure, receptor interaction nature, and etc.) determine the epigenetic outcome.

A particularly poorly studied area is the role of mitochondrial danger signals and mitochondria in innate immune memory formation. Mitochondria represent sites for inflammasome formation, source ROS and DAMPs such as mtDNA, and mediators of metabolic reprogramming during immune cell activation. mtDAMP secretion in response to cellular stress activates PRR-mediated signaling cascades, including NLRP3 inflammasome activation, TLR9 interaction, and cGAS-STING induction (293, 294). It was established that mitochondrial metabolism is critical for epigenetic reprogramming through acetyl-CoA (295). In a recently published work (296), the role of mitochondria as pivotal nodes in integrating metabolism, stress signaling, and innate immunity is discussed. In particular, the authors suggested that physical exercise can be considered as a form of controllable mitochondrial stressor. Temporary mtDAMP, succinate, ROS, and cardiolipin release as a result of physical exercise activates TLR9 and cGAS-STING, SUCNR1 (212), and NLRP3 inflammasome (297) and stabilizes HIF-1α (119). Activation signals promote the shift from oxidative phosphorylation to the aerobic glycolysis through HIF-1α, providing acetyl-CoA and precursor supply for epigenetic modifications. Subsequently, metabolic reprogramming occurs, promoting the shift from the oxidative phosphorylation to the aerobic glycolysis, as well as epigenetic changes (H3K4me3 and H3K27ac), preparing proinflammatory genes for enhanced secondary response. Moderate physical activity promotes immune system homeostasis with low-grade inflammation reduction and reactivity increase, whereas a sedentary lifestyle promotes chronic low-grade inflammation, and excessive high-intensity training may temporarily reduce immune competence.

### 5.5 Trained immunity in organisms with different hypoxia tolerance

Since trained immunity can be induced by mtDNA (288), oxidized LDL (298), catecholamines (299), and hyperglycemia (300), it is possible that the proinflammatory phenotype observed in hypoxia-susceptible organisms may be developed under the influence of various stimuli during ontogenesis. At the same time, in tolerant organisms, the possible tolerance strategy of innate immune cells or balance of tolerance and trained immunity determines less reactivity to stimuli or its absence, i.e., greater tolerance to various stimuli, including hypoxia (protective phenotype). At the same time, there are still no data on moderately hypoxia-tolerant organisms and functional features of their immune cells (**Figure 3**).

**Figure 3:**
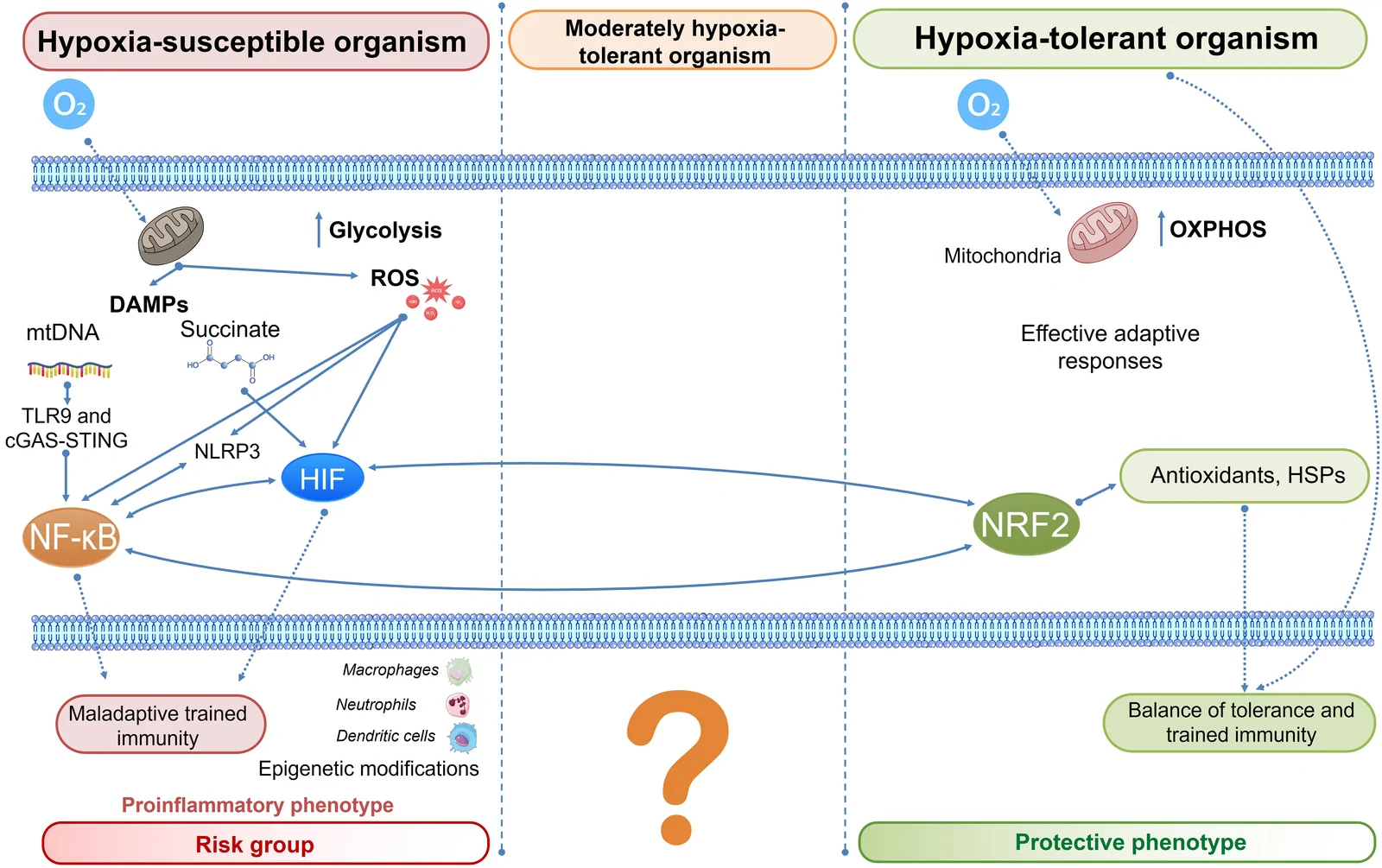
Overview of the suggested mechanisms connecting organism low hypoxia tolerance, mitochondrial dysfunction, and oxidative stress, with the proinflammatory phenotype and maladaptive trained immunity. Balance between HIF, NRF2, and NF-κB determines the outcome (successful adaptative response or maladaptive response) but also depends on organism hypoxia tolerance. Hypoxia-tolerant organisms (right) are characterized by the increased number of small mitochondria in comparison with susceptible ones, reflecting greater OXPHOS intensity. Hypoxia-tolerant organisms are also characterized by a greater number of cristae and high mitochondrial enzyme level. In addition, hypoxia-tolerant organisms exhibit high tolerance to various stimuli, including oxygen deficiency, due to efficient OXPHOS and antioxidant defense activity, which together support successful adaptive responses to oxygen deprivation and the balance of tolerance and trained immunity. This may be associated with the protective phenotype. In contrast, hypoxia-susceptible organisms (left) exhibit constitutively elevated HIF activity, increased glycolysis, and oxidative stress, which are associated with mitochondrial dysfunction. This leads to NF-κB activation and proinflammatory phenotype development and characterizes them as a risk group for the high-altitude and inflammatory diseases. This proinflammatory phenotype and high DAMP release may be associated with the epigenetic modifications in immune cells and maladaptive trained state. Data on moderately hypoxia-tolerant organisms are not presented in the literature. The figure highlights the suggested mechanistic differentiation and emphasizes the need to take into account the individual organism hypoxia tolerance in the hypoxic therapy and high-altitude medicine.

While trained immunity is aimed at pathogen elimination, it may contribute to inflammatory disease progression. In contrast, tolerance may promote recovery/protection mechanisms from some inflammatory pathologies, but on the other hand, its occurrence correlates with an increased risk of secondary infections. Both of these opposite innate immune system responses represent an extremely complex, multilevel process regulated by various signaling pathway changes, chromatin modulation, and metabolic reprogramming (301). Kim et al. (288) note that the main problem in this field is the lack of unified experimental criteria for determining innate immunity memory. Different research groups use different primary stimuli (from LPS to β-glucan and necrotic cell extracts) and different assessment methods. As a result, what is designated as trained immunity in one study may not fully correspond to the conditions of another. There are also contradictory results; for instance, some studies report that tolerant macrophages demonstrate the reduced ability to eliminate pathogens, whereas others report that they retain or even enhance certain antimicrobial functions. These discrepancies indicate unknown modulators (such as cell type, species, or temporal differences) that are not considered. The lack of standardized models is a problem, as without common criteria it is difficult to compare results and build a coherent theory.

Since the key mechanisms of trained immunity are epigenetic changes and metabolic reprogramming, they are also connected with organism hypoxia tolerance and proinflammatory phenotype. Hypoxia-susceptible organisms may initially have “metabolically trained” immunity. Possibly, their immune cells (or their bone marrow precursors) are already in the state corresponding to the trained phenotype: they are metabolically reprogrammed toward enhanced glycolysis, have an altered mitochondrial network, and are epigenetically ready for hyperproduction of proinflammatory cytokines. This phenotype may be a consequence of both genetic and epigenetic factors, microbiota features, diet, or HIF pathway functioning and the microRNAs regulating it. Due to structural and functional mitochondrial features, hypoxia-susceptible organisms’ mitochondria may be more sensitive to stress factors, and release of mtDAMPs—succinate and mtDNA, as well as ROS—under mitochondrial stress in them may be more pronounced. All this leads to basally higher HIF activity, glycolysis, and proinflammatory activation. Temporary mtDAMPs release activates TLR9 and cGAS-STING; succinate activates SUCNR1 (212) and stabilizes HIF-1α (119), increasing IL-1β production (302); and ROS and cardiolipin activate NLRP3 inflammasome (297). These pathways are interconnected with transcription factors NF-κB, NRF2, and HIF-1 activation, thereby mediating redox-based epigenetic modification and proinflammatory cytokine synthesis by immune cells (297, 303, 304). In hypoxia-tolerant organisms, the system likely works differently due to initial high OXPHOS level and antioxidant activity. Additional studies are needed, including assessment of glycolysis, mitochondrial functioning, and epigenetic modifications in animals with different hypoxia tolerance.

**Figure 3** represents the suggested mechanism of proinflammatory phenotype development in hypoxia-susceptible organisms due to high oxidative stress and DAMPs levels, the source of which is mitochondria, and which lead to the development of an epigenetically regulated maladaptive trained state of innate immune cells. To experimentally confirm this mechanism, studies are needed in which mitochondrial respiration parameters, mtDNA levels, glycolysis markers, and epigenetic modifications in macrophages in hypoxia-tolerant and hypoxia-susceptible animals would be simultaneously assessed.

## 6 Conclusion and perspectives

Thus, despite the fact that at the local level the connection of HIF, NRF2, and NF-κB pathways is quite well studied, their role in the development of various diseases, especially those accompanied by the pronounced hypoxia, as well as in individuals living at high altitudes, is still insufficiently investigated. Furthermore, despite sufficient data on genetically, epigenetically, and phenotypically determined variability of individual organism hypoxia tolerance, it is not considered in experimental and clinical studies, as well as in hypoxic therapy. At the same time, according to experimental studies, hypoxia-susceptible organisms are characterized by initially high HIF levels, glycolysis, and a proinflammatory phenotype that determines the severity of inflammatory and tumor diseases. During mountain ascent, as well as when using hypoxic therapy and hypoxic preconditioning, individual organism hypoxia tolerance and the initial proinflammatory phenotype of susceptible organisms should be considered, since incorrect selection of hypoxic exposure dose in them may lead to adverse consequences. Probably, the outcome of innate immune cell functional reprogramming may depend on individual organism hypoxia tolerance. In this regard, additional studies in this scientific field and the development of methods for correcting the proinflammatory phenotype in hypoxia-susceptible organisms for hypoxia tolerance increasing are needed. Therapy strategies for hypoxia-susceptible organisms should be aimed at reducing initially high HIF and glycolysis levels and also include anti-inflammatory agents. In addition, studies of moderately hypoxia-tolerant organisms’ features, as well as adaptive immune cells, whose functioning largely depends on oxygen content, are needed.
